# Force–Thermal Coupling Effects on Surface Integrity and Subsurface Damage of Al-50 wt% Si Alloy During Milling

**DOI:** 10.3390/ma19132885

**Published:** 2026-07-06

**Authors:** Lu Jing, Fengjun Chen, Qiulin Niu, Qiu Hong, Jian Liu, Jiangnan Ding

**Affiliations:** 1 School of Robot Engineering, Wenzhou University of Technology, Wenzhou 325000, China; abccfj@126.com (F.C.); hongqiu@hnu.edu.cn (Q.H.); 20220048@wzut.edu.cn (J.L.); jiangnanding@yeah.net (J.D.); 2 School of Mechanical Engineering, Hunan University of Science and Technology, Xiangtan 411201, China; qlniu2009@163.com

**Keywords:** Al-50 wt% Si alloy, force–thermal coupling, surface integrity, chip morphology, surface defects, subsurface damages

## Abstract

Al-50 wt% Si alloy is widely used in aerospace and electronics but is hard to machine owing to uneven microstructure. To elucidate the relationship between force–thermal coupling effects and surface integrity during Al-50 wt% Si alloy milling, this paper established a stress model to reveal the superposition mechanism of mechanical and thermal stresses. Experiments were conducted to investigate the evolution of cutting forces and temperatures, as well as their influence on surface integrity characteristics, including microhardness, roughness, and chip morphology. The results showed that temperature increases steadily with *v*_c_, whereas cutting force fluctuates in an irregular manner. The maximum cutting temperature rises by 85.04% as *v*_c_ increases from 25 m/min to 125 m/min. Meanwhile, the thermo-mechanical coupling effect exerts a regulatory role on chip morphology, where higher *v*_c_ improves chip continuity and ductility. Surface integrity is determined by the competitive interplay between work hardening and thermal softening, and the surface microhardness varies from 168.49 HV to 173.27 HV. Specifically, elevated *v*_c_ optimizes surface quality, with the *R*a decreasing by 24.54%, whereas excessive *f*_z_ and *a*_p_ aggravate damage. Ultimately, surface defects arise from the combined removal behavior of Si particles and deformation of the Al matrix, while the inhomogeneous stress field induces subsurface damage.

## 1. Introduction

Al-50 wt% Si alloy is a typical particle-reinforced metal matrix composite (PRMMC) with excellent comprehensive properties, including low density, a low thermal expansion coefficient, high wear resistance and good castability [1]. It serves as a key structural material for precision components in the aerospace, automotive engine and electronic packaging fields [2]. However, its microstructure, featured by a high volume fraction of hard and brittle Si particles uniformly distributed in the plastic Al matrix, leads to significant mechanical inhomogeneity, making it a typical hard-to-machine material [3,4].

Hu et al. [5] pointed out that the significant mismatch between the particles and the matrix in terms of elastoplastic behavior and thermal properties gives rise to a severe concentration of stress and temperature during the machining of PRMMCs. This inevitably induces various surface defects on the machined surface, including pits, voids, microcracks, particle fragmentation and surface tearing, which lead to deteriorated surface integrity of the machined workpiece [6,7]. Lu et al. [8,9] also pointed out that under the combined effect of cutting force and cutting temperature, the reinforcement and matrix undergo coordinated deformation, resulting in complex machined surfaces. Therefore, achieving the precision machining of Al-50 wt% Si alloy is challenging.

To reveal the thermo-mechanical cutting mechanism of PRMMCs, scholars have conducted modeling research on cutting force and cutting temperature. Sun et al. [10,11] constructed a constitutive model for SiCp/Al composites that took particle effects into consideration. On this basis, Yin et al. [12] comprehensively accounted for the influences of particle volume fraction, size and aspect ratio on cutting force fluctuation during the machining of PRMMCs and established a turning force model for SiCp/Al composites after analyzing the thermomechanical mechanisms in different cutting zones. For the analytical modeling of cutting temperature in PRMMCs, Xiong et al. [13] developed a predictive model for workpiece cutting temperature in end milling of in situ synthesized TiB_2_/7050Al composites. Yin et al. [14,15] established a model to characterize the rake face temperature of cutting tools during the turning of SiCp/Al composites.

Optimizing the particle removal mode is an important means to improve the machining surface integrity of PRMMCs [16,17,18]. Chen et al. [19] experimentally demonstrated that the machined surface defects of the TiB_2_/2024 composite are mainly smearing and micro-scratches induced by TiB_2_ particles at grain boundaries, as well as micro-pits formed by the plowing of micron-sized TiB_2_ particles. By contrast, the surface defects of the TiB_2_/7075 composite are primarily tails caused by clustered particles, thus resulting in lower surface roughness. Jing et al. [20] argued that the transformation of hard particle removal from pull-out to shearing would help improve the surface topography of PRMMCs. According to Fan et al. [21], the presence of SiC particles induces a high strain gradient, resulting in localized shear deformation. Furthermore, studies show that fracture and fragmentation are the dominant material removal mechanisms for SiC particles in SiCp/Al composites, and microcrack propagation during particle removal is a key factor influencing machined surface quality [22]. Li et al. [23] revealed that cutting speed exerts a remarkable influence on the material removal mechanism of SiCp/Al composites. At low cutting speeds, the complete pull-out of SiC particles occurs, leading to inferior surface quality. At moderate cutting speeds, SiC particles undergo fracture under extrusion action, causing moderate damage to the material. At high cutting speeds, SiC particles are crushed by impact forces, minimizing surface damage. Zhou et al. [24] observed that superior hole wall quality can be achieved in the helical milling of SiCp/Al composites under the processing parameters of high spindle speed, small pitch, and low revolution speed. Pramanik and Zhang [25] pointed out that the interactions between the cutting tool and particles, as well as the fracture and debonding of particles, can lead to localized hardening on the SiCp/Al composites machined surface. Sun et al. [26] examined the influence of particle damage on SiCp/Al composites chip formation, categorizing the chip formation mechanisms into three distinct types: plastic deformation, incomplete gross fracture, and complete gross fracture. Liu et al. [27] investigated the subsurface damage in SiCp/Al composites, identifying particle fracture and cavities as the dominant damage modes, which are induced by mechanical and thermal stresses.

Some studies have also adopted assisted machining technologies to regulate cutting forces and temperatures, thereby improving the material removal behavior of PRMMCs. For Si-Al alloys, Chen et al. [28] suggested that the reduction in cutting force is the key factor responsible for the advantages of ultrasonic vibration cutting in machining Al-50 wt% Si alloy. This technique can reduce the surface roughness of the workpiece and mitigate defects such as voids, cracks, and pits, thereby improving the surface quality. Milling is one of the most commonly used machining processes for components in the aerospace and automotive industries [29,30]. Accordingly, Jing et al. [31] performed groove milling on an Al-50 wt% Si alloy and observed that ultrasonic vibration lowers the average cutting force and temperature, which significantly reduces the fracture and spalling of Si particles and converts their dominant removal mode to shearing. Liu et al. [32] revealed that temperature critically affects both tool adhesion and oxidative wear, with these effects ultimately deteriorating the machined surface quality. Therefore, pulsed laser-assisted turning was employed in the machining of the Al-50 wt% Si alloy. Yu et al. [20,33] demonstrated that compared with dry milling, the scCO_2_-OoWMQL condition can effectively reduce the cutting force and friction at the tool–chip interface, thereby significantly mitigating pit defects on the machined surface of the Al-50 wt% Si alloy.

For SiCp/Al composites, Du et al. [34] demonstrated that ultrasonic elliptical vibration cutting of SiCp/Al composites reduces mechanical stress and suppresses subsurface micro-damage by regulating the fracture behavior of SiC particles. Zhou et al. [35] established a specific-cutting-energy-based criterion to investigate SiCp/Al composites, revealing that surface integrity is primarily governed by the machining method and cutting speed. Under ultrasonic elliptical vibration cutting, the surface defects are prone to being converted into plastic deformation features. Zuo et al. [36] investigated the milling performance of SiCp/Al composites under various green cooling/lubrication strategies. They found that scCO_2_ + OoW significantly reduces cutting temperature and cutting force while effectively mitigating surface defects such as voids and cracks. Zhang et al. [37] revealed that laser-assisted machining enhances the uniformity of surface morphology and subsurface integrity in SiCp/Al composites by promoting a uniform thermal history and a more homogeneous distribution of shear flow stress. Peng et al. [38] developed a laser-ultrasonic elliptical vibration turning method and demonstrated that ultrasonic vibration significantly relieves stress, enhances plastic removal capacity, and effectively suppresses machining damage. Yu et al. [39] found that the synergistic effects of laser-induced plastic deformation and ultrasonic chip fracture reduce the cutting force, yielding an improved surface finish of SiCp/Al composites with a roughness of 172 nm. Wang et al. [40] investigated in situ laser-assisted diamond cutting for the ultra-precision machining of SiCp/Al composites and revealed that this approach reduces cutting force and machining stress by up to 31% and 59.9%, respectively, while suppressing large defects related to particle fracture and pull-out. She et al. [41] proposed a cutting strategy that integrates cryogenic and laser assistance for machining SiCp/Al composites. This technique not only reduces cutting forces but also enhances the interfacial strength between SiC and the Al matrix, thereby suppressing particle debonding.

As summarized above, the mismatch in physico-mechanical properties between reinforced particles and the metal matrix in PRMMCs easily causes stress concentration and heat accumulation, which further trigger diverse surface defects. Exploring the surface integrity of the Al-50 wt% Si alloy under thermo-mechanical coupling is thus of great engineering value. However, existing literature mainly focuses on SiCp/Al composites. Given the substantial differences in matrix composition, reinforcement characteristics and mechanical performance between these two materials, current investigations into the force–thermal coupling behaviors and damage evolution of the Al-50 wt% Si alloy remain insufficient and cannot fully support the development of precision machining technology.

**Figure 1:**
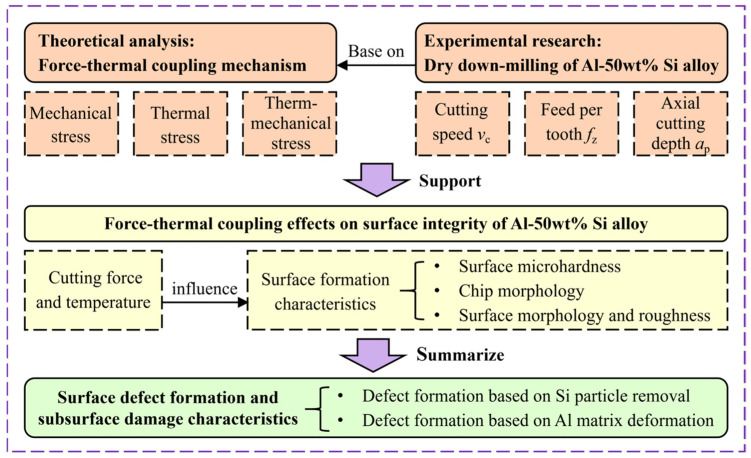
Framework of the overall research approach.

In view of this, this paper carries out a systematic study on the effects of force–thermal coupling on the surface integrity of Al-50 wt% Si alloy during milling. Firstly, a force–thermal coupling stress analysis model dedicated to Al-50 wt% Si alloy milling is established to elucidate the superposition mechanism of mechanical stress induced by cutting force and thermal stress caused by cutting temperature. Secondly, single-factor milling experiments are designed to investigate the influence laws of cutting speed, feed per tooth and axial cutting depth on cutting force and cutting temperature. Then, the surface integrity characteristics including microhardness, chip morphology, surface topography and roughness are systematically characterized. Thirdly, the various types of surface defects dominated by the removal behavior of Si particles and the deformation behavior of the Al matrix are identified, and the subsurface damage characteristics are analyzed. Figure 1 illustrates the overall research framework and methodology. The obtained coupling laws and revealed damage mechanisms are the major novelties of this paper. This work provides theoretical guidance and practical references for the precision milling of Al-50 wt% Si alloy, contributing to process parameter optimization and enhanced machining quality.

## 2. Force–Thermal Coupling Mechanism

During the cutting process of the Al-50 wt% Si alloy, subjected to the combined effect of cutting force and cutting heat, the microstructure of the workpiece surface layer becomes complex. The Al matrix undergoes a series of grain slip and deformation phenomena, while the brittle removal forms of Si particles are diverse. To analyze the action mechanism of cutting force–thermal coupling on the machined surface layer of Al-50 wt% Si alloy, this paper adopts an analytical method to elaborate on the generation of mechanical stress and thermal stress inside the workpiece.

Cutting force (mechanical load) induces elastoplastic deformation and stress concentration in local areas of the material, while cutting temperature (thermal load) causes thermal expansion inside the material. To resist the microstructural deformation caused by both, mechanical stress and thermal stress are generated internally in the material.

### 2.1. Mechanical Stress Induced by Cutting Force

**Figure 2:**
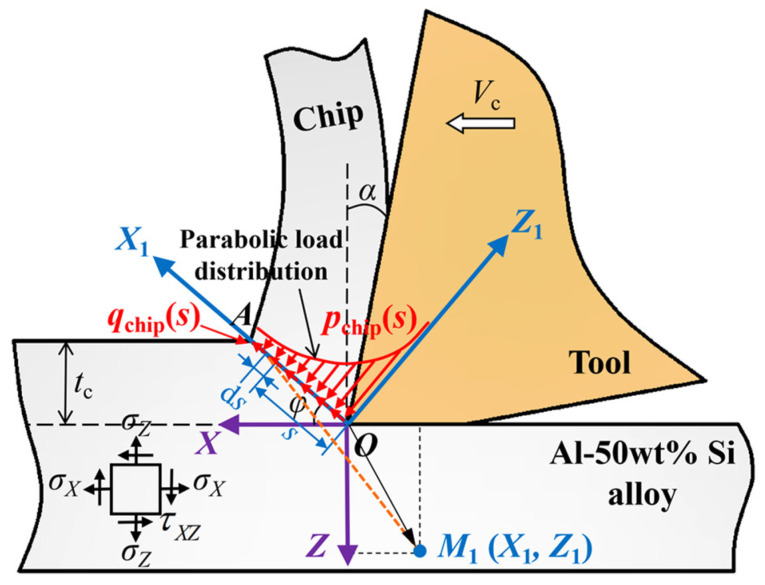
Schematic diagram of mechanical stress distribution caused by chip formation force, adapted from Ref. [27].

Figure 2 shows a schematic diagram of the mechanical stress distribution induced by the chip formation force during cutting. A coordinate system is established as illustrated, where the *X*_1_-axis is parallel to the shear plane *OA* and the *Z*_1_-axis is perpendicular to the shear plane *OA*, with the length of the shear plane denoted as *L*. *p*_chip_(*s*) and *q*_chip_(*s*) represent the normal and tangential load distributions acting on the shear zone, respectively. Since point *O* is closest to the tool edge and thus subjected to the maximum mechanical load, while the load gradually decreases toward point *A*, the mechanical load on the shear plane is assumed to follow a parabolic distribution [27]. The symbol *d*s represents a micro-element on the shear plane at a distance *s* from point *O*, which is subjected to concentrated forces from the two aforementioned mechanical load components. By integrating the Boussinesq solutions for the normal and tangential point loads acting on a semi-infinite body within the contact region, the mechanical stress in the workpiece surface layer induced by the mechanical loads can be obtained. Therefore, the expressions for the mechanical stress at a point *M*_1_(*X*_1_, *Z*_1_) in the workpiece surface layer caused by the chip formation force are given as follows [42]:

(1) $$\left\{\begin{array}{l} \sigma_{X_{1}}^{\mathrm{shear}}(X_{1},Z_{1})=-\frac{2Z_{1}}{\pi }\int_{0}^{L}\frac{p_{\mathrm{chip}}(s){\left(X_{1}-s\right)}^{2}}{{\left[{\left(X_{1}-s\right)}^{2}+Z_{1}^{2}\right]}^{2}}\cdot ds-\frac{2}{\pi }\int_{0}^{L}\frac{q_{\mathrm{chip}}(s)\cdot {\left(X_{1}-s\right)}^{3}}{{\left[{\left(X_{1}-s\right)}^{2}+Z_{1}^{2}\right]}^{2}}\cdot ds\\ \sigma_{Z_{1}}^{\mathrm{shear}}(X_{1},Z_{1})=-\frac{2{Z_{1}}^{3}}{\pi }\int_{0}^{L}\frac{p_{\mathrm{chip}}(s)}{{\left[{\left(X_{1}-s\right)}^{2}+Z_{1}^{2}\right]}^{2}}\cdot ds-\frac{2{Z_{1}}^{2}}{\pi }\int_{0}^{L}\frac{q_{\mathrm{chip}}(s)\cdot (X_{1}-s)}{{\left[{\left(X_{1}-s\right)}^{2}+Z_{1}^{2}\right]}^{2}}\cdot ds\\ \tau_{X_{1}Z_{1}}^{\mathrm{shear}}(X_{1},Z_{1})=-\frac{2{Z_{1}}^{2}}{\pi }\int_{0}^{L}\frac{p_{\mathrm{chip}}(s)(X_{1}-s)}{{\left[{\left(X_{1}-s\right)}^{2}+Z_{1}^{2}\right]}^{2}}\cdot ds-\frac{2Z_{1}}{\pi }\int_{0}^{L}\frac{q_{\mathrm{chip}}(s)\cdot {\left(X_{1}-s\right)}^{2}}{{\left[{\left(X_{1}-s\right)}^{2}+Z_{1}^{2}\right]}^{2}}\cdot ds \end{array}\right.$$

Since the total mechanical stress at a point in the workpiece surface layer is induced by both the chip formation force and the tool flank–workpiece friction, a rotation matrix **Q** is introduced to combine these two stress components. This matrix transforms the mechanical stress at point *M*_1_(*X*_1_, *Z*_1_), calculated in the *O-X*_1_*Z*_1_ coordinate system and caused by the chip formation force, into the *O-XZ* coordinate system, as given in Equations (2) and (3).

(2) $$\left[Q\right]=\left[\begin{array}{l} \cos(\pi -\varphi )\;\;\;\;\sin(\pi -\varphi )\\ -\sin(\pi -\varphi )\;\cos(\pi -\varphi ) \end{array}\right]$$

(3) $$\left[\sigma_{\mathrm{shear}\;X\text{-}Z}\right]=\underset{\mathrm{In}\;\mathrm{the}\;O\text{-}XZ\;\mathrm{coordinate}\;\mathrm{system}}{\underset{︸}{\left[\begin{array}{l} \sigma_{X}^{\mathrm{shear}}\;\tau_{XZ}^{\mathrm{shear}}\\ \tau_{XZ}^{\mathrm{shear}}\;\sigma_{Z}^{\mathrm{shear}} \end{array}\right]}}=\left[Q\right]\underset{\mathrm{In}\;\mathrm{the}\;O\text{-}X_{1}Z_{1}\;\mathrm{coordinate}\;\mathrm{system}}{\underset{︸}{\left[\begin{array}{l} \sigma_{X_{1}}^{\mathrm{shear}}\;\tau_{X_{1}Z_{1}}^{\mathrm{shear}}\\ \tau_{X_{1}Z_{1}}^{\mathrm{shear}}\;\sigma_{Z_{1}}^{\mathrm{shear}} \end{array}\right]}}\left[Q^{T}\right]$$

where $\sigma_{X}^{\mathrm{shear}}$ (*X*, *Z*) and $\sigma_{Z}^{\mathrm{shear}}$ (*X*, *Z*) represent the normal stresses in the *X* and *Z* directions at a point *M*(*X*, *Z*), induced by the chip formation force, respectively; $\tau_{XZ}^{\mathrm{shear}}$ (*X*, *Z*) denotes the shear stress at this point.

**Figure 3:**
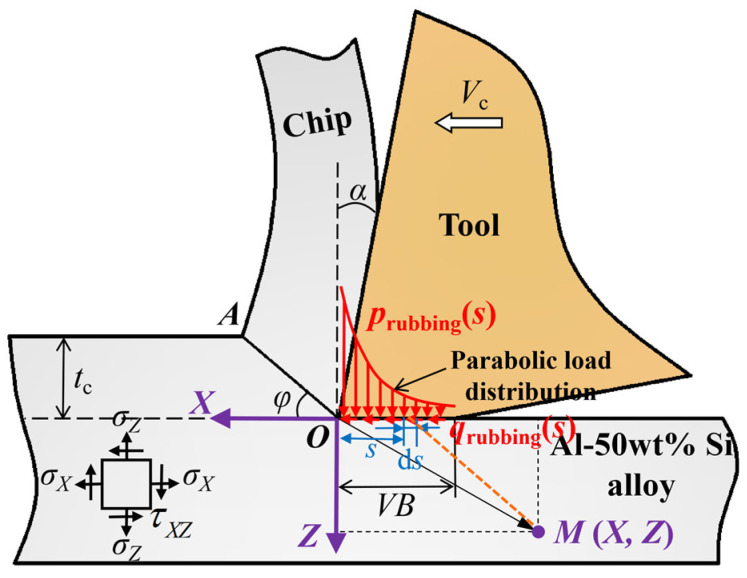
Schematic diagram of mechanical stress distribution caused by friction force, adapted from Ref. [27].

Figure 3 shows the characteristics of the mechanical stress distribution induced by the tool–workpiece friction force, which is regarded as the combined action of a parabolic normal load *p*_rubbing_(*c*) and a tangential load *q*_rubbing_(*c*) acting on the tool flank–workpiece interface. d*c* represents a micro-element on the tool–workpiece contact zone, located at a distance *c* from point *O* and subjected to concentrated forces from the normal and tangential mechanical loads. The expressions for the mechanical stress at point *M*(*X*, *Z*) in the workpiece surface layer induced by the tool–workpiece friction force are as follows:

(4) $$\left\{\begin{array}{l} \sigma_{X}^{\mathrm{rubbing}}(X,Z)=-\frac{2Z}{\pi }\int_{0}^{VB}\frac{p_{\mathrm{rubbing}}(c){\left(X-c\right)}^{2}}{{\left[{\left(X-c\right)}^{2}+Z^{2}\right]}^{2}}\cdot dc-\frac{2}{\pi }\int_{0}^{VB}\frac{q_{\mathrm{rubbing}}(c)\cdot {\left(X-c\right)}^{3}}{{\left[{\left(X-c\right)}^{2}+Z^{2}\right]}^{2}}\cdot dc\\ \sigma_{Z}^{\mathrm{rubbing}}(X,Z)=-\frac{2Z^{3}}{\pi }\int_{0}^{VB}\frac{p_{\mathrm{rubbing}}(c)}{{\left[{\left(X-c\right)}^{2}+Z^{2}\right]}^{2}}\cdot dc-\frac{2Z^{2}}{\pi }\int_{0}^{VB}\frac{q_{\mathrm{rubbing}}(c)\cdot (X-c)}{{\left[{\left(X-c\right)}^{2}+Z^{2}\right]}^{2}}\cdot dc\\ \tau_{XZ}^{\mathrm{rubbing}}(X,Z)=-\frac{2Z^{2}}{\pi }\int_{0}^{VB}\frac{p_{\mathrm{rubbing}}(c)(X-c)}{{\left[{\left(X-c\right)}^{2}+Z^{2}\right]}^{2}}\cdot dc-\frac{2Z}{\pi }\int_{0}^{VB}\frac{q_{\mathrm{rubbing}}(c)\cdot {\left(X-c\right)}^{2}}{{\left[{\left(X-c\right)}^{2}+Z^{2}\right]}^{2}}\cdot dc \end{array}\right.$$

where $\sigma_{X}^{\mathrm{rubbing}}$ (*X*, *Z*) and $\sigma_{Z}^{\mathrm{rubbing}}$ (*X*, *Z*) represent the normal mechanical stresses in the *X* and *Z* directions at a point *M*(*X*, *Z*) of Al-50 wt% Si alloy, induced by the tool–workpiece friction force, respectively; $\tau_{XZ}^{\mathrm{rubbing}}$ (*X*, *Z*) denotes the shear stress at this point caused by the friction force.

By combining Equations (1) and (4), the total mechanical stresses $\sigma_{X}^{\mathrm{mechanical}}$ (*X*, *Z*), $\sigma_{Z}^{\mathrm{mechanical}}$ (*X*, *Z*) and $\tau_{XZ}^{\mathrm{mechanical}}$ (*X*, *Z*) at a point *M*(*X*, *Z*) on the Al-50 wt% Si alloy surface layer can be obtained as follows:

(5) $$\left\{\begin{array}{l} \sigma_{X}^{\mathrm{mechanical}}(X,Z)=\sigma_{X}^{\mathrm{shear}}(X,Z)+\sigma_{X}^{\mathrm{rubbing}}(X,Z)\\ \sigma_{Z}^{\mathrm{mechanical}}(X,Z)=\sigma_{Z}^{\mathrm{shear}}(X,Z)+\sigma_{Z}^{\mathrm{rubbing}}(X,Z)\\ \tau_{XZ}^{\mathrm{mechanical}}(X,Z)=\tau_{XZ}^{\mathrm{shear}}(X,Z)+\tau_{XZ}^{\mathrm{rubbing}}(X,Z) \end{array}\right.$$

### 2.2. Thermal Stress Induced by Cutting Temperature

The increase in cutting temperature will induce the generation of thermal stress inside the workpiece. The expressions for the thermal stress at a point *M*(*X*, *Z*) on the Al-50 wt% Si alloy surface layer are given as follows [43]:

(6) $$\left\{\begin{array}{l} \sigma_{X}^{\mathrm{therm}}(X,Z)=-\frac{\alpha_{w}E_{w}}{1-2\nu_{w}}\int_{0}^{\infty }\int_{-\infty }^{\infty }\left(G_{xh}\frac{\partial T_{\mathrm{Si}\text{-}\mathrm{Al}\;\mathrm{alloy}}^{\mathrm{total}}}{\partial X}\left(X^{\prime },Z^{\prime }\right)+G_{xv}\frac{\partial T_{\mathrm{Si}\text{-}\mathrm{Al}\;\mathrm{alloy}}^{\mathrm{total}}}{\partial Z}\left(X^{\prime },Z^{\prime }\right)\right)\cdot dX^{\prime }dZ^{\prime }+\\ \;\;\;\;\;\;\;\;\;\;\;\;\;\;\;\;\;\;\;\;\;\;\;\;\frac{2Z}{\pi }\int_{-\infty }^{\infty }\frac{g(x){\left(x-X\right)}^{2}}{{\left[{\left(x-X\right)}^{2}+Z^{2}\right]}^{2}}dx-\frac{\alpha_{w}E_{w}T_{\mathrm{Si}\text{-}\mathrm{Al}\;\mathrm{alloy}}^{\mathrm{total}}(X,Z)}{1-2\nu_{w}}\\ \sigma_{Z}^{\mathrm{therm}}(X,Z)=-\frac{\alpha_{w}E_{w}}{1-2\nu_{w}}\int_{0}^{\infty }\int_{-\infty }^{\infty }\left(G_{zh}\frac{\partial T_{\mathrm{Si}\text{-}\mathrm{Al}\;\mathrm{alloy}}^{\mathrm{total}}}{\partial X}\left(X^{\prime },Z^{\prime }\right)+G_{zv}\frac{\partial T_{\mathrm{Si}\text{-}\mathrm{Al}\;\mathrm{alloy}}^{\mathrm{total}}}{\partial Z}\left(X^{\prime },Z^{\prime }\right)\right)\cdot dX^{\prime }dZ^{\prime }+\\ \;\;\;\;\;\;\;\;\;\;\;\;\;\;\;\;\;\;\;\;\;\;\;\;\frac{2Z^{3}}{\pi }\int_{-\infty }^{\infty }\frac{g(x)}{{\left[{\left(x-X\right)}^{2}+Z^{2}\right]}^{2}}dx-\frac{\alpha_{w}E_{w}T_{\mathrm{Si}\text{-}\mathrm{Al}\;\mathrm{alloy}}^{\mathrm{total}}(X,Z)}{1-2\nu_{w}}\\ \tau_{XZ}^{\mathrm{therm}}(X,Z)=-\frac{\alpha_{w}E_{w}}{1-2\nu_{w}}\int_{0}^{\infty }\int_{-\infty }^{\infty }\left(G_{xzh}\frac{\partial T_{\mathrm{Si}\text{-}\mathrm{Al}\;\mathrm{alloy}}^{\mathrm{total}}}{\partial X}\left(X^{\prime },Z^{\prime }\right)+G_{xzv}\frac{\partial T_{\mathrm{Si}\text{-}\mathrm{Al}\;\mathrm{alloy}}^{\mathrm{total}}}{\partial Z}\left(X^{\prime },Z^{\prime }\right)\right)\cdot dX^{\prime }dZ^{\prime }+\\ \;\;\;\;\;\;\;\;\;\;\;\;\;\;\;\;\;\;\;\;\;\;\;\;\frac{2Z^{2}}{\pi }\int_{-\infty }^{\infty }\frac{g(x)(x-X)}{{\left[{\left(x-X\right)}^{2}+Z^{2}\right]}^{2}}dx \end{array}\right.$$

(7) $$g(x)=\frac{\alpha_{w}E_{w}T_{\mathrm{Si}\text{-}\mathrm{Al}\;\mathrm{alloy}}^{\mathrm{total}}(X=Z=0)}{1-2\nu_{w}}$$

where $\sigma_{X}^{\mathrm{therm}}$ (*X*, *Z*) and $\sigma_{Z}^{\mathrm{therm}}$ (X, Z) represent the thermal stresses in the *X* and *Z* directions at point *M*(*X*, *Z*), respectively; $\tau_{XZ}^{\mathrm{therm}}$ (*X*, *Z*) denotes the thermal shear stress at this point, acting on the *X*-plane in the *Z* direction. $\alpha_{w}$ and $\nu_{w}$ represent the coefficient of thermal expansion and Poisson’s ratio of the workpiece, and *g*(*x*) is the stress load induced by the surface temperature.

*G*_xh_, *G*_xv_, *G*_zh_, *G*_zv_, *G*_xzh_, and *G*_xzv_ are the plane-strain Green’s functions, which describe the strain field distribution induced by a unit point force on an infinite plane and represent the relationship between the strain induced by the unit point force and the position within the field. The plane-strain Green’s function is generally expressed as *G*(*x*, *y*, *x*′, *y*′), where *x* and *y* are the coordinates of the field point, and *x*′ and *y*′ are the coordinates of the source point on the plane. It describes the strain field distribution induced by a unit point force applied at the source point (*x*′, *y*′). By evaluating the plane-strain Green’s function at different positions (*x*, *y*) on the plane, the distribution of the strain field across the entire plane induced by the unit point force can be obtained. The expressions for these functions are given by Saif et al. [43].

### 2.3. Effect of Thermo-Mechanical Stress on Machined Surface

During the cutting process of the Al-50 wt% Si alloy, the total stress at point *M*(*X*, *Z*) on the workpiece surface layer is the sum of the mechanical stress and thermal stress at this location, as expressed in Equation (8):

(8) $$\left\{\begin{array}{l} \sigma_{X}(X,Z)=\sigma_{X}^{\mathrm{shear}}(X,Z)+\sigma_{X}^{\mathrm{rubbing}}(X,Z)+\sigma_{X}^{\mathrm{therm}}(X,Z)\\ \sigma_{Z}(X,Z)=\sigma_{Z}^{\mathrm{shear}}(X,Z)+\sigma_{Z}^{\mathrm{rubbing}}(X,Z)+\sigma_{Z}^{\mathrm{therm}}(X,Z)\\ \sigma_{Y}(X,Z)=\nu_{w}\left[\sigma_{X}(X,Z)+\sigma_{Z}(X,Z)\right]-\alpha_{w}E_{w}T_{\mathrm{Si}\text{-}\mathrm{Al}\;\mathrm{alloy}}^{\mathrm{total}}(X,Z)\\ \tau_{XZ}(X,Z)=\tau_{XZ}^{\mathrm{shear}}(X,Z)+\tau_{XZ}^{\mathrm{rubbing}}(X,Z)+\tau_{XZ}^{\mathrm{therm}}(X,Z)\\ \tau_{XY}(X,Z)=\tau_{YZ}(X,Z)=0 \end{array}\right.$$

where $\sigma_{X}$ (*X*, *Z*), $\sigma_{Z}$ (*X*, *Z*), and $\sigma_{Y}$ (*X*, *Z*) represent the total normal stresses at point *M* in the *X*, *Y*, and *Z* directions, respectively. $\tau_{XZ}$ (*X*, *Z*), $\tau_{XY}$ (*X*, *Z*), and $\tau_{YZ}$ (*X*, *Z*) are the total shear stresses at this point.

Under the action of the inhomogeneous stress field induced by the cutting force–thermal coupling, the surface layer of the Al-50 wt% Si alloy undergoes deformation and failure.

## 3. Milling Experiments

### 3.1. Experimental Design

**Figure 4:**
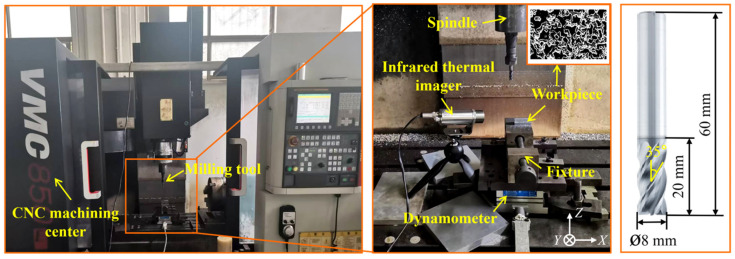
Milling experimental setup.

Figure 4 shows the experimental setup employed for the end milling of an Al-50 wt% Si alloy on a VMC-850 three-axis vertical machining center with dry down-milling. The milling force acquisition system consists of a three-component piezoelectric dynamometer (Type 9257B, Kistler Instrumente AG, Winterthur, Switzerland), an 8-channel NI9205 data acquisition card, and a charge amplifier. An Optris PI infrared thermal imager (Optris GmbH & Co. KG, Berlin, Germany) was separately employed to measure the cutting temperature. Together, these two sets of equipment were used to track the variations in cutting force and cutting temperature during the entire milling process. The Al-50 wt% Si alloy workpiece had geometric dimensions of 35 × 16 × 12 mm and was fabricated by spray deposition. The chemical composition as well as the mechanical properties of the material are presented in Table 1 and Table 2, respectively. AlTiN-coated cemented carbide four-flute flat end mills with a diameter of 8 mm were employed, whose detailed specifications are shown in Figure 4.

The cutting tool was clamped using a standard elastic collet tool holder matching the spindle interface of the VMC-850 machining center, which provides sufficient clamping stiffness and positioning precision to ensure stable milling conditions during testing. The rectangular workpiece was rigidly clamped inside a precision machine vise. This vise was securely mounted and bolted onto the upper surface of the three-axis dynamometer, and the whole dynamometer assembly was fixed to the worktable of the machining center to suppress workpiece vibration and positional drift in the milling process. For the three force measurement directions defined on the dynamometer, the X-axis corresponds to the worktable moving horizontally rightward, the Y-axis is perpendicular to the X-axis and points inward, and the Z-axis is collinear with the machine spindle and oriented vertically upward.

The milling test plan is presented in Table 3. The radial cutting depth *a*_e_ was fixed at 4 mm, while the cutting speed *v*_c_, feed per tooth *f*_z_, and axial cutting depth *a*_p_ were adopted as single-factor variables. For each group of experiments, a new tool was used to eliminate the impact of tool wear. After milling, the workpiece was cleaned using an ultrasonic cleaner.

### 3.2. Experimental Equipment and Methods

Based on the evolution of cutting force and cutting temperature, the surface hardening, chip morphology, surface morphology, and surface roughness of the Al-50 wt% Si alloy were investigated. In addition, the subsurface damage and surface defect characteristics during milling were analyzed, together with their formation mechanisms.

#### 3.2.1. Surface Roughness Measurement

**Figure 5:**
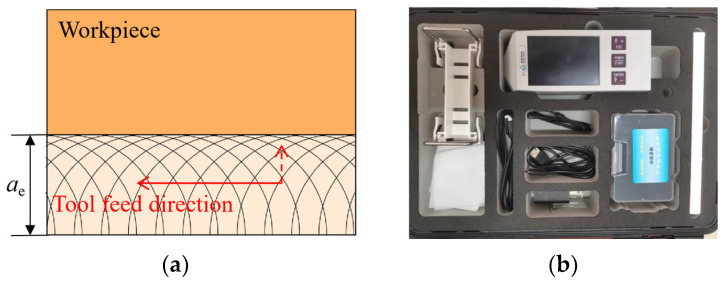
Surface roughness measurement: (**a**) Directions of surface roughness measurement; (**b**) Surface roughness tester.

The arithmetic mean deviation *R*a and the ten-point height of micro-irregularities *R*z were selected as surface roughness evaluation parameters for the machined profile. The measurement method is illustrated in Figure 5a. For each set of cutting parameters, measurements were taken six times along the tool feed direction (X-direction) and perpendicular to the feed direction (Y-direction), respectively, and the average values were adopted. Figure 5b shows the JITAI820 surface roughness tester (Beijing Jitai Tech Detection Device Co., Ltd., Beijing, China).

#### 3.2.2. Surface Hardness Measurement

**Figure 6:**
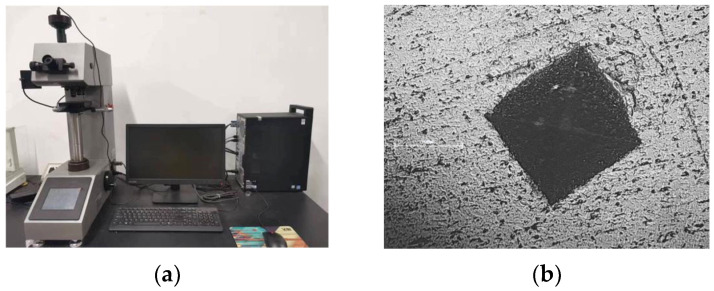
Surface hardness measurement: (**a**) Vickers hardness tester; (**b**) indentation of a positive quadrangular indenter.

The microhardness of the Al-50 wt% Si alloy machined surface was measured using a SHYCHVT-30Z Vickers hardness tester (Shanghai Hongce Instrument Technology Co., Ltd., Shanghai, China) with an applied load of 5 kgf and a dwell time of 10 s. Measurement points were selected along the center of the machined surface, and the average value was obtained from six measurements. Figure 6a and Figure 6b show the hardness testing equipment and the indentation of the square pyramid indenter, respectively.

#### 3.2.3. Subsurface Sample Preparation

**Figure 7:**
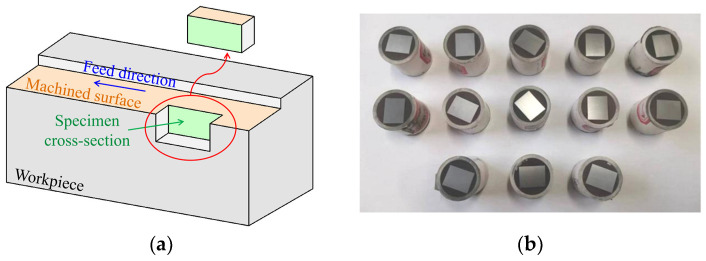
Sampling and preparation of subsurface specimens: (**a**) diagram of sampling; (**b**) inlaid specimens.

A cross-section perpendicular to the machined surface was taken to characterize subsurface damage, and the sampling schematic is shown in Figure 7a. Figure 7b shows the subsurface specimens of the Al-50 wt% Si alloy. They were prepared by cold mounting, in which dental powder and resin were uniformly mixed and poured into PVC tubes. Grinding was performed after the resin was fully cured. The specimens were ground sequentially using sandpapers of grit sizes 220#, 400#, 600#, 800#, 1000#, 1500#, and 2000# to obtain a fine and uniform surface finish. Subsequently, the specimens were polished with diamond suspension polishing fluid, etched using Keller’s reagent, rinsed with anhydrous ethanol, and dried to characterize the subsurface damage induced by Al-50 wt% Si alloy milling.

Scanning electron microscopy (SEM) was employed to characterize the chip morphology, machined surface and subsurface of the Al-50 wt% Si alloy. Meanwhile, energy dispersive spectroscopy (EDS) was performed at selected locations to determine the elemental species and content at these sites.

## 4. Results and Discussion

### 4.1. Cutting Force and Temperature

**Figure 8:**
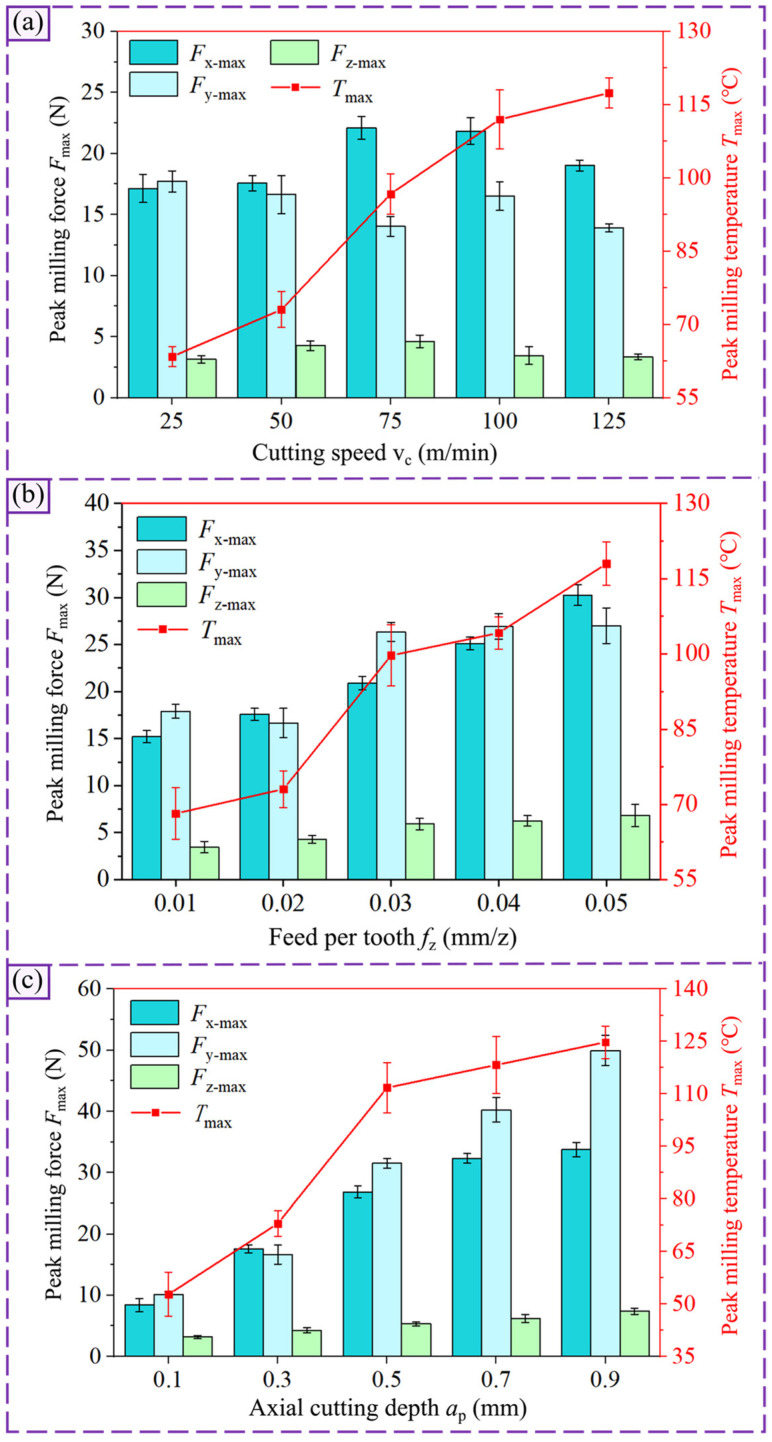
Effect of cutting parameters on milling force and temperature: (**a**) variations under different cutting speeds; (**b**) variations under different feed per tooth; (**c**) variations under different axial cutting depths.

Figure 8 shows the effect of cutting parameters on the peak milling force *F*_max_ and peak temperature *T*_max_ during the Al-50 wt% Si alloy milling. Figure 8a presents the variation trends of the three-component milling forces and milling temperature with *v*_c_. It can be observed that the three-component peak milling forces *F*_x-max_, *F*_y-max_, and *F*_z-max_ exhibit irregular fluctuations with increasing *v*_c_. The cutting temperature rises with increasing *v*_c_. The maximum cutting temperature under a cutting speed of *v*_c_ = 125 m/min is 85.04% higher than that measured at *v*_c_ = 25 m/min. This measured trend conforms to the thermal stress evolution law predicted by the force–thermal coupling model. Increasing *v*_c_ improves the material removal rate, and enhances the generation of shear heat and friction heat, consequently leading to greater thermal stress within the workpiece.

As shown in Figure 8b, both the milling force and temperature of the Al-50 wt% Si alloy increase with increasing *f*_z_. The increase in *f*_z_ leads to a larger cutting thickness and material removal rate, which raises the number of hard particles and the Al matrix contact area on the tool, thus increasing the chip formation force, tool–chip friction, and cutting temperature. When *f*_z_ increases from 0.01 mm/z to 0.05 mm/z, *F*_x-max_, *F*_y-max_, and *F*_z-max_ increase by 99.01%, 50.84%, and 98.15%, respectively; meanwhile, *T*_max_ increases by 73.02%. The effect of *a*_p_ on *F*_max_ and *T*_max_ is shown in Figure 8c. As the increase in *a*_p_ significantly raises the material removal volume and enhances the chip formation force, both *F*_max_ and *T*_max_ increase with the rise in *a*_p_. At *a*_p_ = 0.9 mm, *F*_x-max_, *F*_y-max_, and *F*_z-max_ increase by 301.40%, 392.22% and 132.32%, respectively, compared with those at *a*_p_ = 0.1 mm; *T*_max_ increases by 136.36%.

### 4.2. Surface Formation Characteristics

**Figure 9:**
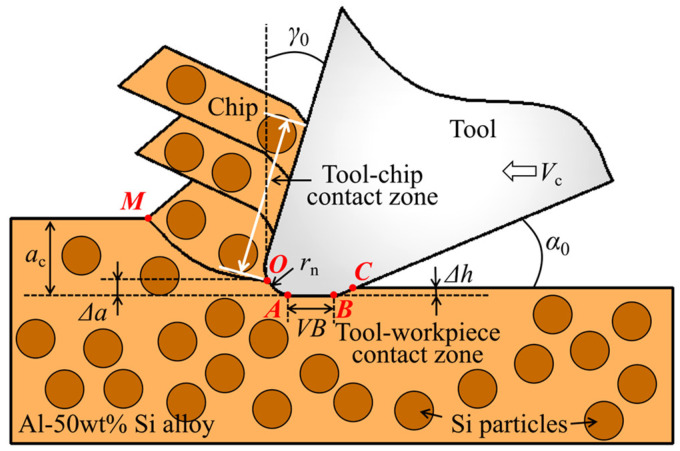
Machined surface formation in Al-50 wt% Si alloy cutting.

As shown in Figure 9, during the actual machining of the Al-50 wt% Si alloy, due to the existence of the tool cutting edge radius *r*_n_ and the flank wear *VB*, the entire cutting layer thickness *a*_c_ cannot be completely removed. There is always an extremely thin layer of workpiece material Δ*a* that fails to slide along the shear slip line *OM*. Instead, it is extruded and deformed by the tool cutting edge at the separation point *O*. As the removal mechanisms of the matrix phase and the reinforced phase in the Al-50 wt% Si alloy are significantly different, Si particles are prone to fracture, pull-out, or being carried away by chips under the action of the cutting tool, resulting in various defects on the machined surface. Meanwhile, the tool flank is constantly scratched by partially fractured, broken, or detached Si particles, which aggravate the abrasive wear of the tool. The Al matrix possesses strong plastic deformability. It rebounds instantaneously by a height of Δ*h* after machining, making the machined surface slightly higher than the cutting line. This causes the surface-layer material in segment *BC* to continue rubbing against the tool flank.

Therefore, the tool–workpiece contact length is the sum of the cutting edge radius segment *OA*, the flank wear width segment *AB*, and the elastic recovery segment *BC* of the matrix. During the cutting process, the tool–workpiece contact zone exerts normal pressure and tangential friction on the workpiece surface, resulting in elastic–plastic deformation of the Al-50 wt% Si alloy. Consequently, a modified layer featuring work hardening and increased surface roughness is formed, accompanied by surface defects and subsurface damage.

#### 4.2.1. Surface Microhardness

**Figure 10:**
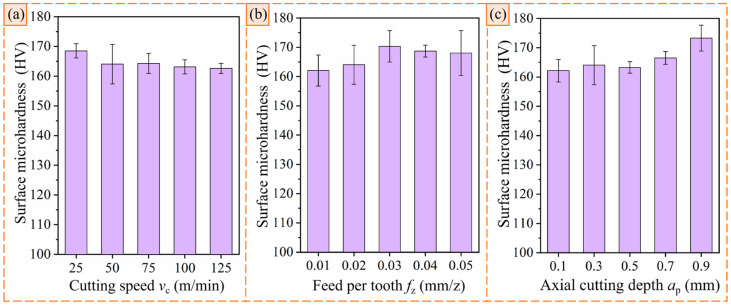
Effects of cutting parameters on the surface microhardness: (**a**) cutting speed; (**b**) feed per tooth; (**c**) axial cutting depth.

Figure 10 shows the variation trend of the microhardness of the machined surface with cutting parameters. During the Al-50 wt% Si alloy milling, under the action of the cutting force, elastic–plastic deformation occurs in the third deformation zone, which produces a strengthening effect on the workpiece surface, namely, work hardening. Meanwhile, the cutting heat generated by the plastic deformation of the Al matrix and the friction of Si particles will soften the Al matrix and reduce the material strength. Therefore, variations in machined surface microhardness arise from the competing strain hardening and thermal softening behaviors governed by the superimposed mechanical and thermal stress field defined in the force–thermal coupling model.

Figure 10a shows the effect of *v*_c_ on the surface microhardness. It can be seen that the surface hardness exhibits an overall downward trend with increasing *v*_c_. At *v*_c_ = 25 m/min, the surface hardness reaches a maximum of 168.49 HV. With a continuous increase in *v*_c_, the surface hardness decreases to a minimum at *v*_c_ = 125 m/min. This is because the cutting force does not change significantly with increasing *v*_c_, and the shorter contact time of the material in the cutting zone leads to insufficient plastic deformation, thereby reducing the work hardening degree of the Al-50 wt% Si alloy. Meanwhile, the cutting temperature rises with increasing *v*_c_, which further weakens the work hardening effect. In addition, owing to the relatively high volume fraction of Si particles in the workpiece, as well as their random size and distribution, the work hardening effect on the machined surface is relatively weak.

As shown in Figure 10b, the microhardness of the machined surface first increases and then decreases with the increase in *f*_z_. The maximum microhardness of 170.32 HV is obtained at *f*_z_ = 0.03 mm/z. This is because the increase in *f*_z_ enhances the deformation degree of the machined surface layer, leading to an increase in hardness. When *f*_z_ exceeds a certain value, the effect of cutting temperature gradually becomes dominant, resulting in a slight decrease in surface hardness.

Figure 10c shows that the surface hardness exhibits an overall upward trend with the increase in *a*_p_. The maximum surface hardness of 173.27 HV is achieved at *a*_p_ = 0.9 mm. This is because the tool–workpiece contact area and cutting force per unit time are positively correlated with *a*_p_, which increases the deformation degree of the material surface and thus improves the hardness. Although the cutting temperature also rises with the increase in *a*_p_, the proportion of the Al matrix in the Al-50 wt% Si alloy is relatively low. Under the thermo-mechanical coupling effect, the final surface hardness still shows an increasing trend.

#### 4.2.2. Chip Morphology

**Figure 11:**
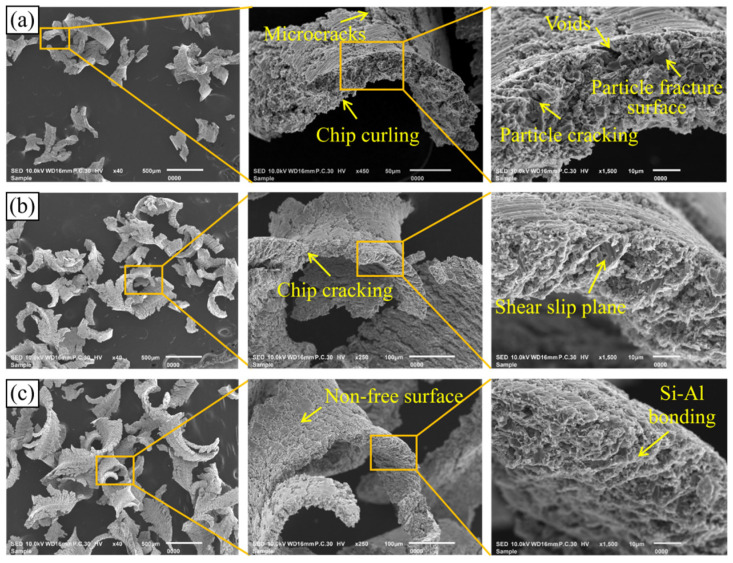
Effect of cutting speed on chip morphology: (**a**) *v*_c_ = 25 m/min, (**b**) *v*_c_ = 75 m/min, (**c**) *v*_c_ = 125 m/min.

Through the analysis of chip morphology, the material removal process can be indirectly evaluated. Figure 11 shows the effect of *v*_c_ on the chip morphology during the milling of the Al-50 wt% Si alloy. It can be seen that with the increase in *v*_c_, the chip length increases and the degree of serration decreases, indicating improved chip continuity and ductility. This can be attributed to the thermal softening effect of the Al matrix. As *v*_c_ increases, the cutting temperature rises, which promotes the plastic flow of the Al matrix. This reduces the dislocation density generated between the Al matrix and Si particles during shear slip, thereby decreasing the number and propagation of microcracks [44].

As shown in Figure 11a, obvious microcracks appear on the non-free surface of the chips when *v*_c_ = 25 m/min. The extrusion deformation inside the material results in microstructural features such as voids caused by particle fracture and debonding. Owing to the large number of Si particles, the chips are generally brittle, and some chips exhibit cracking, as illustrated in Figure 11b. As depicted in Figure 11c, when *v*_c_ increases to 125 m/min, the non-free surface of chips becomes relatively smooth, and the chips show a relatively uniform mixed-bonded state in which the Al matrix encapsulates broken particles. This is attributed to the enhanced plastic deformation of the Al matrix. In addition, the friction and extrusion at the tool–chip interface raise the temperature in the contact zone, which affects the chip curl degree.

**Figure 12:**
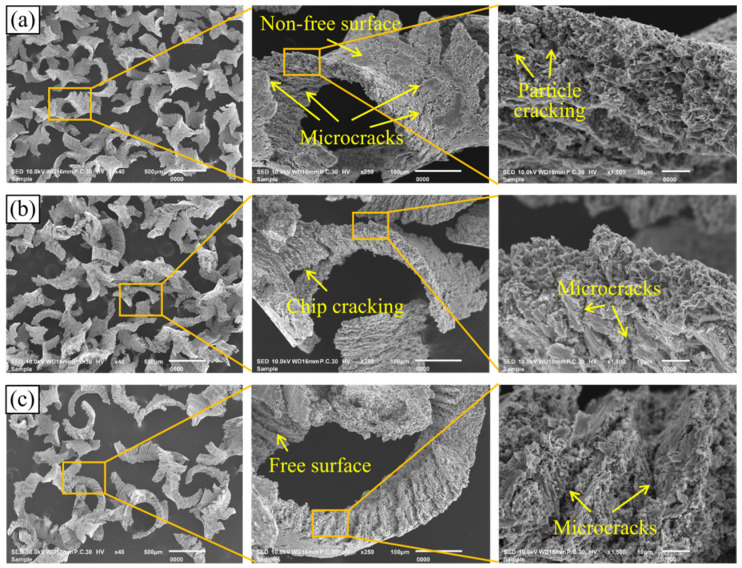
Effect of feed per tooth on chip morphology: (**a**) *f*_z_ = 0.01 mm/z, (**b**) *f*_z_ = 0.03 mm/z, (**c**) *f*_z_ = 0.05 mm/z.

Figure 12 shows the chip morphology under different *f*_z_. Macroscopically, a smaller *f*_z_ leads to a smaller chip cross-sectional area, making it easier for internal defects such as microcracks and voids to coalesce and propagate through the chip, resulting in chip breakage. Therefore, the chips are the finest when *f*_z_ = 0.01 mm/z. As observed in Figure 12a, morphologies such as microcracks and particle cracking can be seen. With the increase in *f*_z_, the instantaneous cutting thickness increases, making it more difficult for microcracks initiated and propagated from the chip-free surface to penetrate through to the non-free surface. Another possible reason is that the increase in *f*_z_ raises the material deformation and the tool–chip contact area, leading to a higher cutting temperature and enhanced plastic deformation capability of the Al matrix [45]. These two factors contribute to the improvement of chip continuity. Therefore, as shown in Figure 12c, the longest chips are obtained at *f*_z_ = 0.05 mm/z. Due to the material anisotropy, the chips exhibit a distinct irregular serrated morphology.

**Figure 13:**
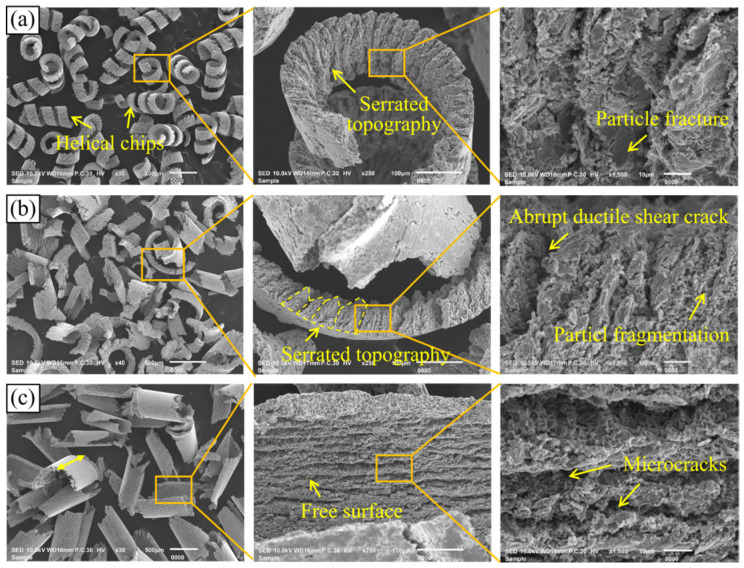
Effect of cutting depth on chip morphology: (**a**) *a*_p_ = 0.1 mm, (**b**) *a*_p_ = 0.5 mm, (**c**) *a*_p_ = 0.9 mm.

Figure 13 shows the effect of *a*_p_ on the chip morphology. As shown in Figure 13a, the chip presents a helical macro-morphology at *a*_p_ = 0.1 mm. Microstructural observations indicate that the chip-free surface is serrated. This is attributed to the relatively small tool–chip contact area and cutting force under this condition, allowing the chip to flow smoothly along the cutting edge and thus form a helical shape. However, due to the presence of Si particles, the material deformation remains non-uniform, and characteristics such as particle fracture and fragmentation can be observed on the chip-free surface. The chip morphology at *a*_p_ = 0.5 mm is shown in Figure 13b. Under this condition, the macroscopic helical chips are no longer obvious, while the chips still exhibit a serrated morphology at the microscale, with non-periodic shear cracks observable. As illustrated in Figure 13c, when *a*_p_ reaches 0.9 mm, both the chip width and tool–chip contact area are maximized, leading to increased cutting force, cutting temperature, and shear slip. Macroscopically, C-shaped chips are formed, and the degree of chip serration is pronounced at the microscopic level.

#### 4.2.3. Surface Morphology and Roughness

**Figure 14:**
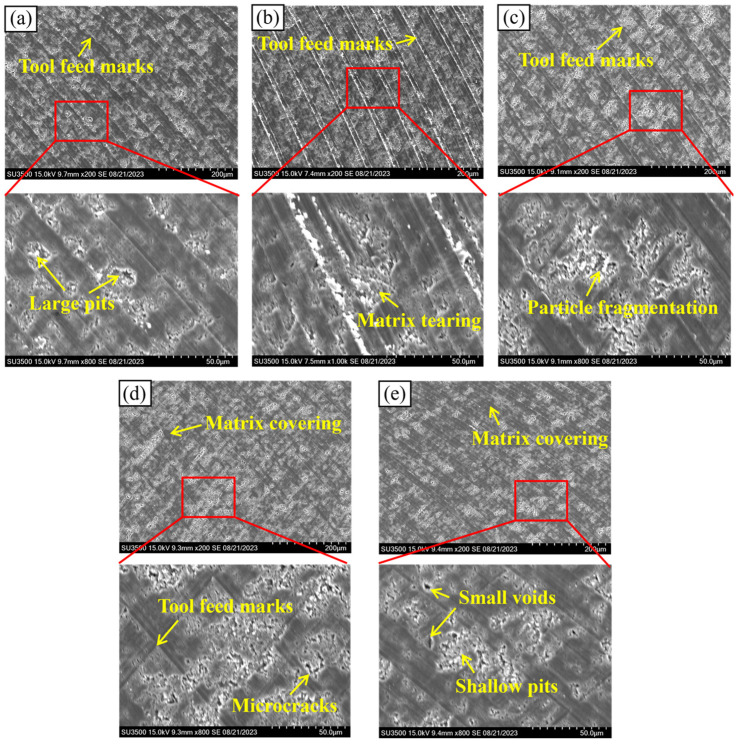
Effect of cutting speed on machined surface morphology: (**a**) *v*_c_ = 25 m/min, (**b**) *v*_c_ = 50 m/min, (**c**) *v*_c_ = 75 m/min, (**d**) *v*_c_ = 100 m/min, (**e**) *v*_c_ = 125 m/min.

Figure 14 shows the machined surface morphology of the Al-50 wt% Si alloy under the effect of *v*_c_. As can be seen from Figure 14a,b, at *v*_c_ = 25 m/min and 50 m/min, obvious tool feed marks are present on the machined surface, together with large pits caused by Si particle pull-out and fragmentation, accompanied by Al matrix tearing due to tool–workpiece friction and extrusion. As shown in Figure 14d,e, with increasing *v*_c_ up to 100 m/min and 125 m/min, the material removal mode of Si particles gradually changes from spalling and fracture to dominant shearing, eventually leading to surface characteristics such as microcracks and shallow pits. This can be attributed to the increased strain rate imposed on the particles by the cutting tool at higher *v*_c_. As the cutting temperature rises with increasing *v*_c_, the failure mode of the Al matrix transforms from tearing damage to relatively uniform matrix covering, and the tool feed marks are significantly weakened. In addition, small void defects are observed at *v*_c_ = 125 m/min, which may result from the weakened interfacial bonding strength between particles and the softened matrix induced by the elevated cutting temperature, thus causing local particle debonding. Similar conclusions have been reported by Szwajka et al. [46], who verified that the microstructural evolution of 316 L stainless steel is correlated with the variation in cutting parameters.

**Figure 15:**
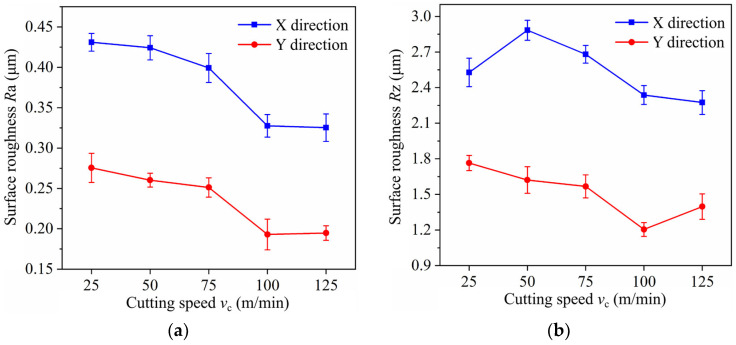
Effect of cutting speed on machined surface roughness: (**a**) *R*a in the X and Y directions, (**b**) *R*z in the X and Y directions.

Figure 15 presents the evolution of surface roughness with *v*_c_. It can be seen that both *R*a and *R*z in the X and Y directions of the machined surface exhibit a decreasing trend with increasing *v*_c_. This is because a higher *v*_c_ facilitates the shear fracture of Si particles and reduces the number of cavities induced by particle pull-out. Meanwhile, the increased cutting temperature per unit time leads to the softening of the Al matrix, which further reduces *R*a and *R*z. The highest cutting temperature is achieved at *v*_c_ = 125 m/min, where the softened Al matrix may weaken the holding effect and the interfacial bonding strength between the matrix and particles, slightly increasing the probability of particle debonding and pull-out. Consequently, *R*a and *R*z show a slight fluctuation compared with those at *v*_c_ = 100 m/min. In addition, the surface roughness in the X direction is consistently higher than that in the Y direction, indicating that the measured surface roughness is dependent on the measuring direction. The *R*a values along the X and Y directions of the machined surface at *v*_c_ = 125 m/min are 0.325 μm and 0.195 μm, respectively, which are reduced by 24.54% and 29.31% compared with those at *v*_c_ = 25 m/min.

**Figure 16:**
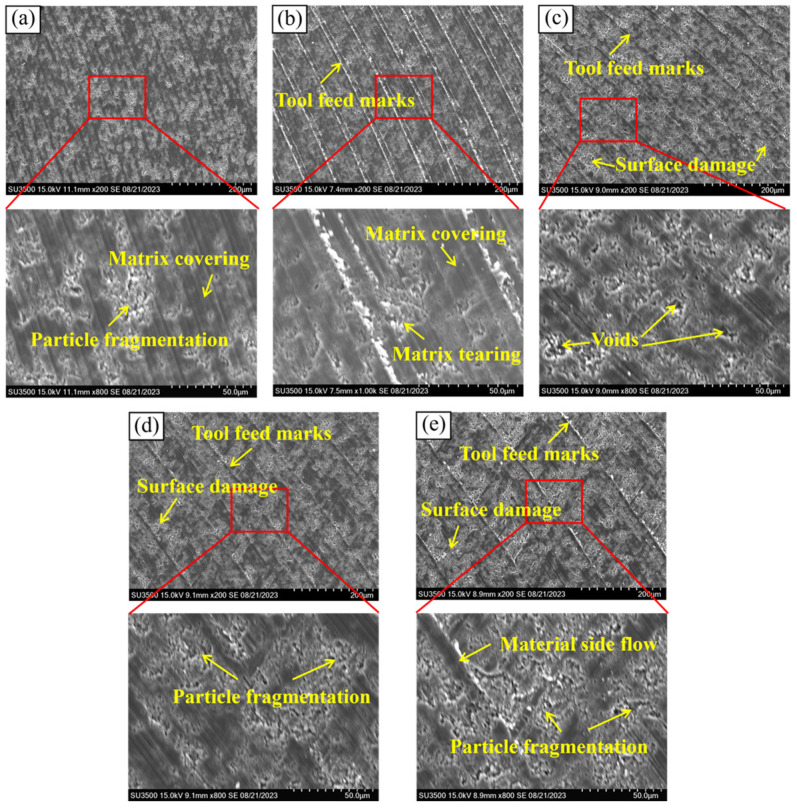
Effect of feed per tooth on machined surface morphology: (**a**) *f*_z_ = 0.01 mm/z, (**b**) *f*_z_ = 0.02 mm/z, (**c**) *f*_z_ = 0.03 mm/z, (**d**) *f*_z_ = 0.04 mm/z, (**e**) *f*_z_ = 0.05 mm/z.

Figure 16 shows the machined surface morphology under the effect of *f*_z_. As can be seen from Figure 16a,b, at *f*_z_ = 0.01 mm/z and 0.02 mm/z, the material removal rate and cutting force per unit time are relatively low. This results in a relatively uniform matrix covering on the machined surface, and the surface damage is dominated by small-area fragmentation. As shown in Figure 16c,d, the tool load and cutting force gradually increase with increasing *f*_z_, and microcracks tend to propagate through the Si particles, leading to cleavage fracture of the particles. Consequently, the area of surface damage caused by particle fragmentation and pull-out in Al-50 wt% Si alloy is enlarged at *f*_z_ = 0.03 mm/z and 0.04 mm/z. Meanwhile, according to tool kinematics, an increase in *f*_z_ lengthens the trajectory span of the cutting edge, and the existence of the end cutting edge inclination angle increases the material residual height. Therefore, as shown in Figure 16e, material side flow and tool feed marks are most significant on the surface, and the surface damage is the most severe when *f*_z_ = 0.05 mm/z.

**Figure 17:**
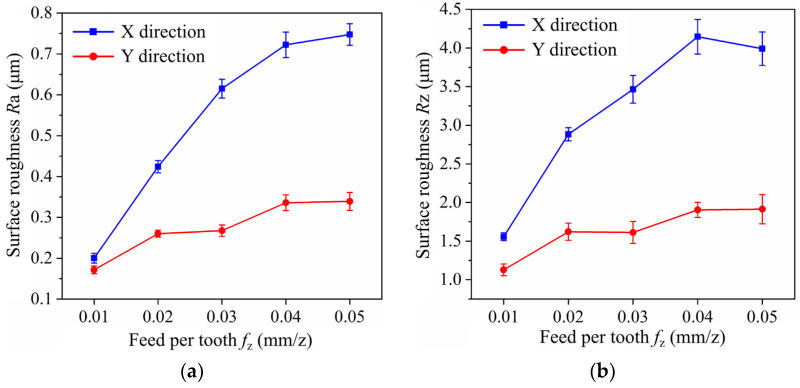
Effect of feed per tooth on machined surface roughness: (**a**) *R*a in the X and Y directions, (**b**) *R*z in the X and Y directions.

Figure 17 presents the evolution of surface roughness with *f*_z_. The roughness values *R*a and *R*z in both the X and Y directions of the machined surface exhibit an increasing trend with the increase in *f*_z_. When *f*_z_ is in the range of 0.01–0.04 mm/z, the surface roughness increases sharply with increasing *f*_z_. This is attributable to the fact that the increase in *f*_z_ raises the peak-to-valley height difference in the machined surface profile. Meanwhile, the increased cutting force aggravates surface defects and deteriorates the surface quality. When *f*_z_ reaches 0.05 mm/z, the surface roughness changes slightly. This is because when *f*_z_ increases to a certain value, although the machined surface damage of the Al-50 wt% Si alloy is the most severe, the characteristics of the surface defects do not change significantly. The *R*a values in the X and Y directions at *f*_z_ = 0.05 mm/z are 0.748 μm and 0.339 μm, respectively, representing increases of 273.13% and 97.57% compared with those at *f*_z_ = 0.01 mm/z.

**Figure 18:**
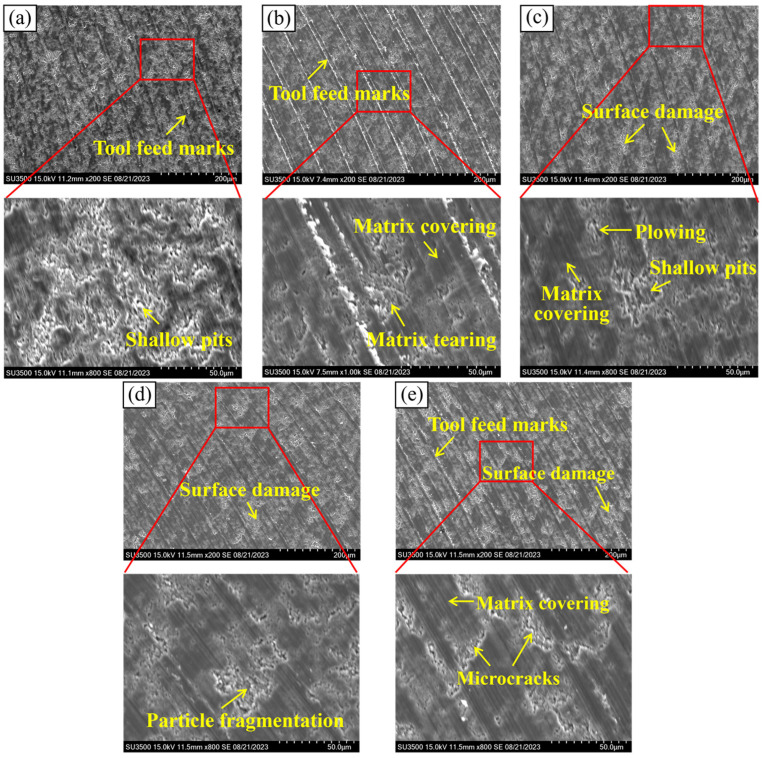
Effect of axial cutting depth on machined surface morphology: (**a**) *a*_p_ = 0.1 mm, (**b**) *a*_p_ = 0.3 mm, (**c**) *a*_p_ = 0.5 mm, (**d**) *a*_p_ = 0.7 mm, (**e**) *a*_p_ = 0.9 mm.

Figure 18 shows the machined surface morphology under the effect of *a*_p_. The interaction force and cutting temperature between the tool–workpiece increase with increasing *a*_p_, leading to greater material flow and deformation in the cutting direction and deteriorated surface morphology. As shown in Figure 18a, the surface damage is dominated by shallow pits resulting from particle fragmentation when *a*_p_ = 0.1 mm. The softening effect of the Al matrix is enhanced with increasing *a*_p_, improving the matrix covering behavior. The machined surface at *a*_p_ = 0.9 mm presented in Figure 18e reveals microcracks beneath the matrix covering layer. In practice, the increase in *a*_p_ intensifies detrimental behaviors such as particle fragmentation, pull-out, and plowing, leading to a larger area of surface damage covered by the matrix.

**Figure 19:**
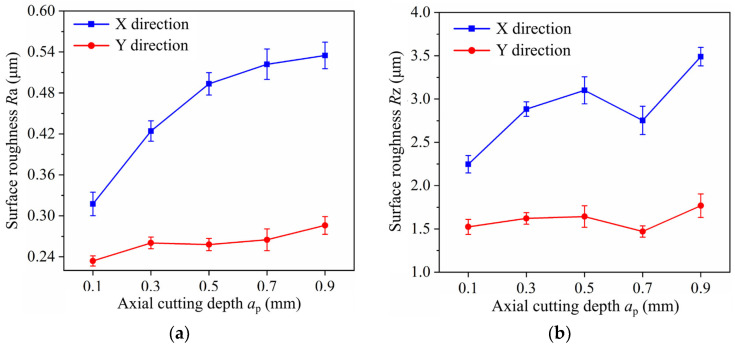
Effect of axial cutting depth on machined surface roughness: (**a**) *R*a in the X and Y directions, (**b**) *R*z in the X and Y directions.

As shown in Figure 19, surface roughness exhibits an upward trend with the increase in *a*_p_. When *a*_p_ = 0.9 mm, the *R*a in the X and Y directions are 0.535 μm and 0.286 μm respectively, which are 69.07% and 22.22% higher than those at *a*_p_ = 0.1 mm.

## 5. Surface Defect Formation

This section summarizes ten surface defects of the Al-50 wt% Si alloy in milling, with their formation processes discussed in detail via SEM and EDS.

### 5.1. Defect Formation Based on Si Particle Removal

**Figure 20:**
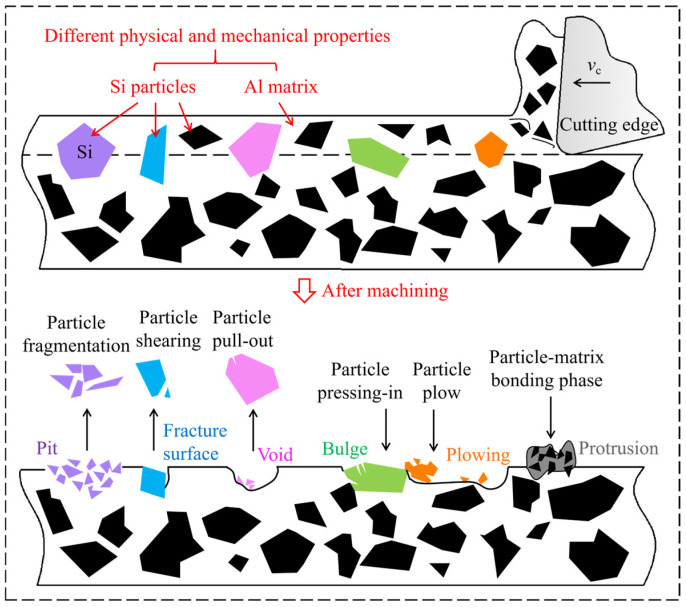
Removal behavior of hard Si particles and corresponding surface defects.

Figure 20 illustrates the main removal modes of hard Si particles, including fragmentation, shearing, pull-out, and pressing-in, each corresponding to specific surface defects such as pits and voids. Subsequently, the effects of various particle removal modes and their derivative behaviors on the Al-50 wt% Si alloy machined surface are discussed separately.

**Figure 21:**
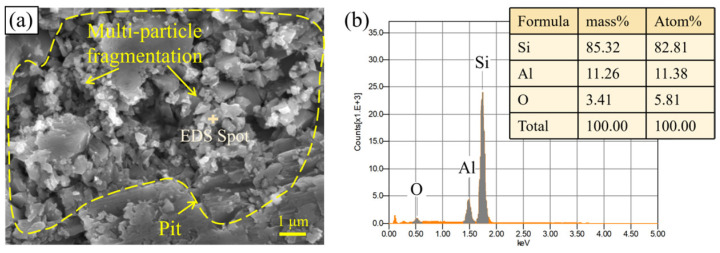
Particle fragmentation and its energy spectrum analysis: (**a**) SEM image; (**b**) EDS spectrum.

Particle fragmentation is the most common mode of particle removal during the Al-50 wt% Si alloy machining. When Si particles are subjected to direct contact from the tool or compression and obstruction by other particles, a high stress concentration is induced. The particles undergo cleavage fracture when the stress reaches their fracture strength. Figure 21 shows the pit defects induced by particle fragmentation and the corresponding EDS results on the Al-50 wt% Si alloy machined surface. It can be seen from Figure 21a that, owing to the high content and dense distribution of Si particles, multiple particles fracture. A large number of crushed fine particles remain on the machined surface, forming pit defects. As shown in Figure 21b, EDS was employed to analyze the fractured particles inside the pits for verification. The results indicate that the constituent elements at this location are Si, Al, and O. Among them, Si accounts for the highest mass fraction of 85.32%, and the small amount of O is caused by slight oxidation on the surface.

**Figure 22:**
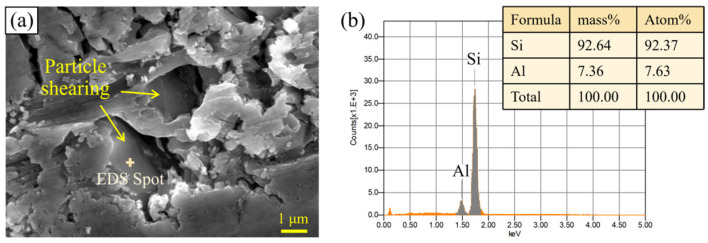
Particle shearing and its energy spectrum analysis: (**a**) SEM image; (**b**) EDS spectrum.

When the Si particle has similar volumes above and below the cutting path, a large aspect ratio, and high particle-matrix interfacial bonding strength, it is prone to brittle shearing under the impact of the cutting edge. It can be observed from Figure 22a that the fracture surface of the Si particle is relatively smooth. This particle removal mode generally helps improve the machined surface morphology. The EDS results in Figure 22b show that the residual sheared Si particles remain well embedded in the Al matrix. At high cutting speeds, the Al matrix cannot undergo sufficient plastic deformation, which facilitates the occurrence of Si particle shearing.

**Figure 23:**
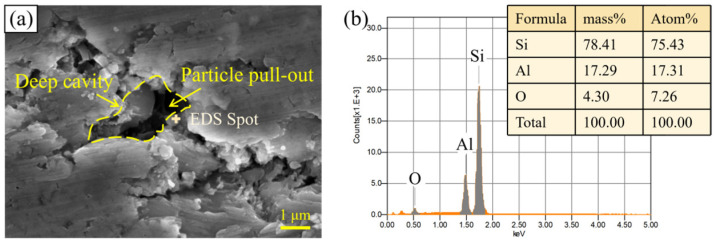
Particle pull-out and its energy spectrum analysis: (**a**) SEM image; (**b**) EDS spectrum.

When the volume of the Si particle above the cutting path is larger than that below, the particle is easily pulled out due to the weak supporting effect of the underlying Al matrix. Figure 23a shows the cavity defects formed by particle pull-out on the milled surface of an Al-50 wt% Si alloy. The size and depth of the cavities are related to the dimensions of the pulled-out particles, and such defects deteriorate the machined surface quality. The EDS results in Figure 23b show that fractured Si particles exist around the cavities.

**Figure 24:**
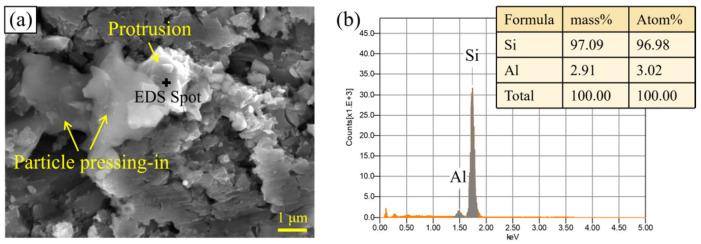
Particle pressing-in and its energy spectrum analysis: (**a**) SEM image; (**b**) EDS spectrum.

When the volume of the Si particle above the cutting path is smaller than that below, the Al matrix beneath provides strong support for the particle. Thus, the particle is easily pressed into the machined surface by the cutting edge, forming protrusion defects. Meanwhile, fractured or pulled-out particles that fall between the flank face and the machined surface may also be pressed in to form protrusions. Figure 24a shows the surface morphology formed by particle pressing-in, exhibiting protrusion characteristics. The EDS results in Figure 24b indicate that the mass fraction of Si at the protrusion is 97.09%, confirming that it is a Si particle.

**Figure 25:**
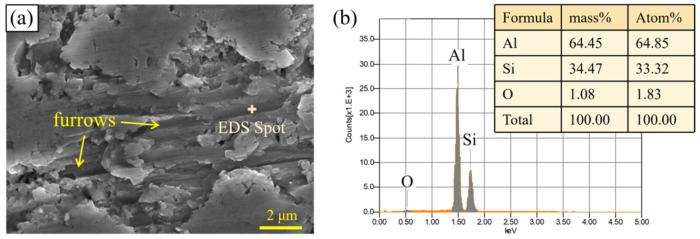
Surface furrows and their energy spectrum analysis: (**a**) SEM image; (**b**) EDS spectrum.

Figure 25a shows the furrow defects on the Al-50 wt% Si alloy machined surface. The formation mechanism can be described as follows: the cutting tool drives particles to plow the surface. Alternatively, fractured and detached Si particles roll and fall into the space between the tool flank face and the machined surface. Under the rotation and extrusion of the milling tool, these particles are pressed into the machined surface and slide along it, thus forming furrow defects. As shown in Figure 25b, since the plowing process mainly occurs on the plastic Al matrix, the mass fraction of Al at the furrow reaches the highest value of 64.45%.

**Figure 26:**
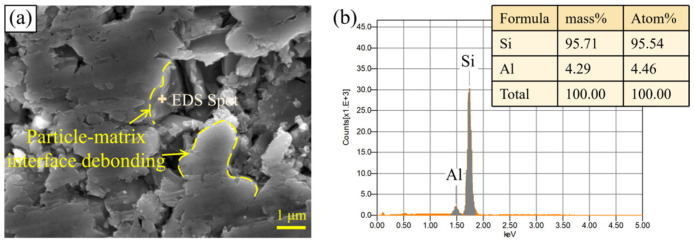
Particle debonding and its energy spectrum analysis: (**a**) SEM image; (**b**) EDS spectrum.

When the stress exerted by the tool on the Si particles exceeds the particle-matrix interfacial strength, particle debonding occurs as shown in Figure 26a. EDS analysis was conducted at the particle-matrix interface in Figure 26b. The results confirm that the particle has completely debonded from the Al matrix, with only 4.29% Al by mass remaining attached to the interface.

**Figure 27:**
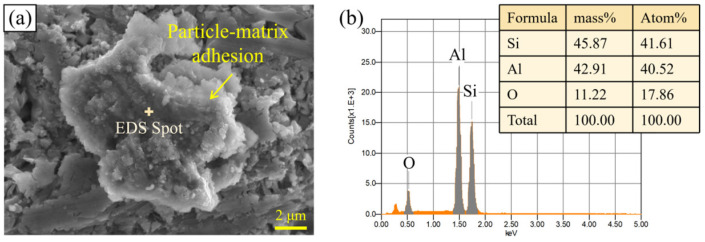
Particle-matrix adhesions and their energy spectrum analysis: (**a**) SEM image; (**b**) EDS spectrum.

Figure 27a shows particle-matrix adhesions attached to the machined surface, resulting in protrusion defects. During the cutting process, numerous broken micro-particles exist on the machined surface. Combined with the plastic deformation and thermal softening effect of the Al matrix, these components easily form Si-Al adhesions under the friction and extrusion between the tool and workpiece. EDS analysis was performed on the adhesion, and the elemental composition is presented in Figure 27b. The mass fractions of Al and Si are 42.91% and 45.87%, respectively, indicating that the contents of Al and Si are comparable and the Si-Al bonding is relatively uniform and sufficient.

### 5.2. Defect Formation Based on Al Matrix Deformation

**Figure 28:**
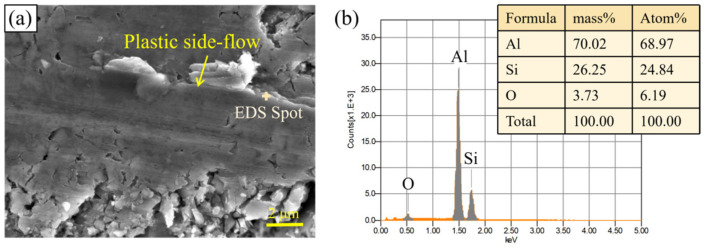
Plastic side-flow and its energy spectrum analysis: (**a**) SEM image; (**b**) EDS spectrum.

Figure 28 shows the plastic side-flow morphology of the Al-50 wt% Si alloy machined surface. During milling, the plastic Al matrix is subjected to the coupled effects of high pressure and cutting heat at the tool tip, leading to expansion and plastic side-flow along the tool flank. This distorts the residual height of the machined surface, deepens the tool feed marks, and thus increases the surface roughness. As observed from Figure 28a, the high content of Si particles in the Al-50 wt% Si alloy results in the presence of broken micro-particles in the side-flow material. A large number of Si particles are fractured under the extrusion of the cutting tool. As shown in Figure 28b, EDS was employed to analyze the side-flow material. The results indicate that the mass fraction of Al is the highest at 70.02%, which is attributed to the fact that the side-flow behavior is dominated by the Al plastic deformation. Si is distributed in the Al matrix with a mass fraction of 26.25%.

**Figure 29:**
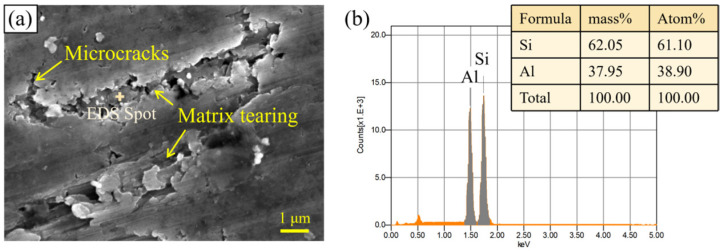
Matrix tearing and its energy spectrum analysis: (**a**) SEM image; (**b**) EDS spectrum.

Figure 29a presents the morphology of microcracks and matrix tearing. The matrix tearing is mainly caused by friction and extrusion between the tool flank face and the machined surface. Tearing occurs when the friction force on the Al matrix is large and the stress under thermo-mechanical coupling reaches its ultimate strength. EDS analysis in Figure 29b reveals that various defects exist directly below the torn Al matrix, which is composed of Al and Si elements with different mass fractions.

**Figure 30:**
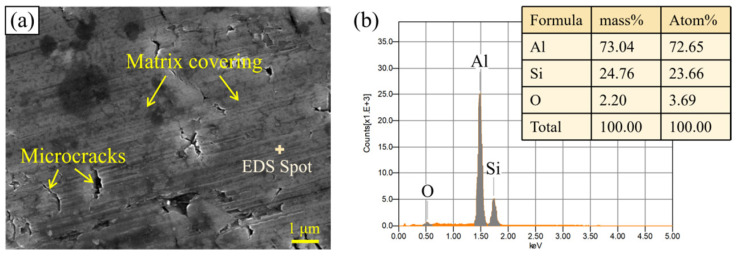
Matrix covering and its energy spectrum analysis: (**a**) SEM image; (**b**) EDS spectrum.

Figure 30a shows the matrix covering morphology. The relatively soft Al matrix is smoothly smeared and covers the machined surface under the thermo-mechanical coupling effect of the tool, filling defects such as pits and voids, which can reduce the surface roughness. However, the covering layer is thin and exhibits poor compactness. It bonds with the defects through weak mechanical adhesion and thus tends to detach easily. It can be observed that microcracks and voids still exist on the machined surface despite the matrix covering. Figure 30b shows that the surface covering layer is dominated by Al with a mass fraction of 73.04%, and Si is attached to it.

## 6. Subsurface Damage Characteristics

**Figure 31:**
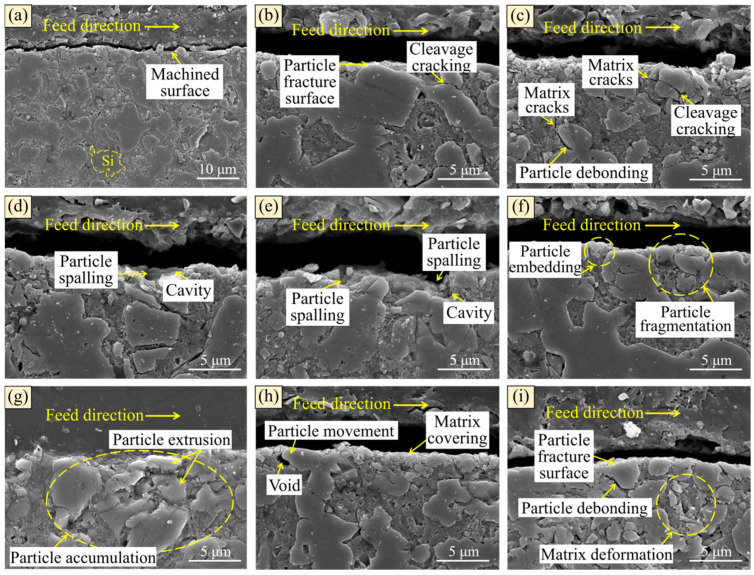
Subsurface damage characteristics in Al-50 wt% Si alloy milling: (**a**) intact Si particles; (**b**) particle fracture and cleavage cracking; (**c**) matrix cracks, particle debonding and cleavage cracking; (**d**) particle spalling and cavity; (**e**) severe particle spalling and cavity; (**f**) particle embedding and particle fragmentation; (**g**) particle extrusion and particle accumulation; (**h**) particle movement, void and matrix covering; (**i**) particle fracture, particle debonding and matrix deformation.

Figure 31 summarizes the subsurface damage characteristics of the Al-50 wt% Si alloy after milling, including particle damage modes such as Si particle cracking, shearing, spalling, embedding, debonding, and accumulation, as well as matrix damage modes such as Al matrix cracking and deformation. Each damage mode and its formation mechanism are discussed separately below. Figure 31a shows the subsurface microstructure of the milled Al-50 wt% Si alloy. It can be seen that due to the interactions among the cutting tool, Si particles, and Al matrix during the cutting process, the machined surface undergoes severe deformation, and corresponding damage occurs in the subsurface.

### 6.1. Particle Damage Characteristics

Figure 31b,c show the cleavage cracking of Si particles. The formation mechanisms can be divided into two types: one is that the particles are cracked by direct contact with the cutting tool, and cracks initiate and propagate in the subsurface; the other is that the particles inside the subsurface are subjected to stress from the matrix or surrounding particles, thus leading to crack formation. Particle shearing can be observed in Figure 31b,i, characterized by relatively smooth fracture surfaces of Si particles. Figure 31d,e illustrate the subsurface damage induced by particle spalling. Cavity defects left on the surface layer after particle removal can be observed, and this type of damage should be minimized during machining. Figure 31f presents particle fragmentation caused by direct contact between the tool–particles. Meanwhile, particles embedded in the subsurface under tool extrusion tend to induce matrix cracking or further fragmentation through interactions with other particles. Figure 31g shows the accumulation behavior of Si particles, which is caused by dislocation pile-up due to elastic–plastic deformation of the surface layer material or uneven particle distribution in local regions. Under this condition, the surface hardness of the workpiece is increased, and the interaction between particles is intensified [47]. Particle debonding can be attributed to two formation mechanisms. Firstly, as shown in Figure 31h,i, the direct action of the tool on the particles causes particle displacement and interfacial debonding, resulting in voids between the particles and the surrounding matrix. Secondly, as illustrated in Figure 31c, particles in the subsurface region undergo debonding under the stress induced by dislocation formation.

### 6.2. Matrix Damage Characteristics

Al matrix cracking is a critical feature of subsurface damage in Al-50 wt% Si alloy cutting, as shown in Figure 31c. Matrix cracking occurs when the stress applied to the matrix exceeds its ultimate strength. When the crack propagates to the Si particles, three interaction mechanisms can be observed: First, the particles hinder further crack propagation, and larger particles tend to suppress the growth of matrix cracks more effectively [48,49,50]. Second, particle debonding occurs when the stress concentration induced by the crack at the particle exceeds the particle-matrix interfacial bonding strength. Third, the matrix crack propagates through the particles, leading to particle cracking, as the stress concentration at the particle boundary reaches the fracture stress of the Si particles. Figure 31i illustrates plastic deformation of the matrix in the subsurface, which induces the flow of fine particles.

## 7. Conclusions

This paper focused on the force–thermal coupling effect during Al-50 wt% Si alloy milling, established a thermo-mechanical coupling stress model, and carried out milling experiments. It systematically revealed the influence rules and formation mechanisms of cutting parameters on cutting force, cutting temperature, surface integrity, surface defects and subsurface damage. The conclusions were as follows.

(1) The coupled mechanical and thermal loads collectively determined the stress field distribution within the surface layer, which further governed the material deformation and removal behavior during Al-50 wt% Si alloy milling.
(2) The surface microhardness was dominated by the competition between work hardening and thermal softening. It decreased with increasing *v*_c_, first increased and then decreased with increasing *f*_z_, and kept rising with increasing *a*_p_. Thermal softening played a leading role at high *v*_c_, while work hardening dominated at large *a*_p_.
(3) Cutting parameters obviously affected chip continuity and ductility. Increasing *v*_c_ enhanced the thermal softening of the Al matrix, thus improving chip continuity and reducing serration and microcracks. Enlarging *f*_z_ and *a*_p_ increased chip section and shear deformation, leading to longer chips but more severe serration and internal defects.
(4) Raising *v*_c_ optimized surface morphology, weakened tool feed marks, restrained particle pull-out and matrix tearing, and effectively reduced surface roughness. Excessive *f*_z_ and *a*_p_ aggravated Si particle fragmentation, pull-out and plowing, resulting in higher roughness and worse surface damage.
(5) Surface defects were induced by the combined effects of Si particle removal and Al matrix deformation. The main removal modes of Si particles included fragmentation, shearing, pull-out, pressing-in, plowing and debonding, corresponding to pits, voids, protrusions and furrows. The Al matrix underwent plastic side-flow, tearing and covering under force–thermal coupling.
(6) Subsurface damage was caused by the inhomogeneous stress field of force–thermal coupling. Particle-related damage included cracking, shearing, spalling, embedding, accumulation and debonding, while matrix damage was dominated by cracking and plastic deformation.

This study clarified the force–thermal coupling mechanism and damage evolution law of the Al-50 wt% Si alloy during milling, providing a theoretical basis and technical support for parameter optimization and precision machining of high Si-Al alloys in electronic packaging fields. This work adopted dry single-factor milling tests and simplified the Si particle distribution in the stress model, which has certain limitations. Future research will concentrate on developing hybrid auxiliary machining processes to reduce machining defects and constructing a multi-objective parameter optimization model for industrial applications.

## Figures and Tables

**Table 1:** Main chemical composition of Al-50 wt% Si alloy.

Component	Si	Fe	Others (Each)	Total Impurities	Al
Content (wt%)	51.37%	0.04%	<0.05%	<0.15%	Balance

**Table 2:** Physical and mechanical properties of Al-50 wt% Si alloy, adapted from Ref. [20].

Properties	Value
Coefficient of thermal expansion (ppm/°C)	11.5
Thermal conductivity (W/mK) at 25 °C	140
Density (g/cm^3^)	2.5
Strength of extension (MPa)	220
Yield strength (MPa)	210
Modulus of elasticity (GPa)	108

**Table 3:** Experimental plan for Al-50 wt% Si alloy milling.

Cutting Speed *v*_c_ (m/min)	Feed per Tooth *f*_z_ (mm/z)	Axial Cutting Depth *a*_p_ (mm)
25/50/75/100/125	0.02	0.3
50	0.01/0.02/0.03/0.04/0.05	0.3
50	0.02	0.1/0.3/0.5/0.7/0.9

## Data Availability

The original contributions presented in this study are included in the article. Further inquiries can be directed to the corresponding author.

## References

[1] Zheng Z., Chen D., Huang K., Zhang J., Wang H., Chen X., Xiao J., Xu J. (2024). Modeling and analysis of surface integrity transition in cutting of Sip/Al composites based on coordination deformation effect of particle-matrix. Tribol. Int..

[2] Li X., Wang L., Chai L., Jin X., Xu C., Du J., Zhang Y., Xue W. (2025). Fabrication and properties of microarc oxidation coatings on Al-50Si alloy with ultra-high Si content under different voltages. J. Alloys Compd..

[3] Liao Z., Abdelhafeez A., Li H., Yang Y., Diaz O.G., Axinte D. (2019). State-of-the-art of surface integrity in machining of metal matrix composites. Int. J. Mach. Tools Manuf..

[4] Wu F., Zhang N., Peng W., Sun Y., Li X., Wang Z. (2023). A novel hybrid micro-texture for improving the wear resistance of PCD tools on cutting SiCp/Al composites. J. Manuf. Process..

[5] Hu C., Zhu Y., Fan R. (2024). Experimental studies of the machinability of SiCp/Al with different volume fractions under ultrasonic-assisted grinding. Materials.

[6] Fu B., Gu Y., Lin J., Zhao J., Liu Y., Luan Y., Li S., Chen J. (2026). Mechanism of the coupling effect of pulse laser remelting and ultrasonic vibration in suppressing surface/subsurface defects and improving wear resistance of Al-50Si. Tribol. Int..

[7] Chen Z., Shu D., Ding F., Chen M., Wang B. (2026). A novel hybrid machining approach for SiCp/Al composites: Insights into material removal mechanisms and surface quality improvement. Ceram. Int..

[8] Lu S., Zhang J., Li Z., Zhang J., Wang X., Hartmaier A., Xu J., Yan Y., Sun T. (2021). Cutting path-dependent machinability of SiCp/Al composite under multi-step ultra-precision diamond cutting. Chin. J. Aeronaut..

[9] Sheng P., Chen Y., Sun W., Cui Y., Xiong D. (2024). Investigation on cutting deformation behavior of Al/SiCp composites considering particle feature distributions. J. Manuf. Process..

[10] Sun W., Duan C., Yin W. (2020). Development of a dynamic constitutive model with particle damage and thermal softening for Al/SiCp composites. Compos. Struct..

[11] Sun W., Duan C., Yin W. (2021). Modeling of force and temperature in cutting of particle reinforced metal matrix composites considering particle effects. J. Mater. Process. Technol..

[12] Yin W., Duan C., Li Y., Miao K. (2021). Dynamic cutting force model for cutting SiCp/Al composites considering particle characteristics stochastic models. Ceram. Int..

[13] Xiong Y., Wang W., Jiang R., Lin K. (2018). Analytical model of workpiece temperature in end milling in-situ TiB_2_/7050Al metal matrix composites. Int. J. Mech. Sci..

[14] Yin W., Duan C., Sun W., Wen B. (2020). Analytical model of cutting temperature for workpiece surface layer during orthogonal cutting particle reinforced metal matrix composites. J. Mater. Process. Technol..

[15] Peng J., Xu Z., Wu T., Zhao B., Wang L., Ding W. (2025). Cutting characteristics and wear mechanisms of SiCp/Al composites machined by PCD milling cutters with varied negative rake angles. J. Manuf. Process..

[16] Xiang D., Cheng Z., Yuan Z., Li Y., Peng P., Song C., Zhang Z., Gao G., Cui X., Tong J. (2024). Multidimensional ultrasonic vibration machining modeling of SiCp/Al cutting forces with different volume fractions: Experiments and numerical simulations. Compos. Commun..

[17] Wang J., Chen G., Wang S., Hou Y., Xu J., Yu H. (2025). Study on the removal behavior of constituent phases of SiCp/Al composites by ultrasonic vibration-assisted milling. Mater. Today Commun..

[18] Sun W., Cheng Z., Wang Y., Liu D., Sheng P., Liu K., Chen J., Li P. (2026). Heterogeneity-induced ductile-brittle transition behavior in negative rake angle cutting of Al/SiCp composites. J. Mater. Process. Technol..

[19] Chen J., Yu W., Zuo Z., Li Y., Chen D., An Q., Geng J., Chen M., Wang H. (2021). Effects of in-situ TiB_2_ particles on machinability and surface integrity in milling of TiB_2_/2024 and TiB_2_/7075 Al composites. Chin. J. Aeronaut..

[20] Jing L., Niu Q., Dang J., An Q., Wang C., Zou F., Li C., Li P., Yue W., Ko T.J. (2022). Milling performance evaluation and cooling/lubrication mechanism of Al-50wt% Si alloy based on various environmentally sustainable manufacturing strategies. Int. J. Adv. Manuf. Technol..

[21] Fan Y., Xu Y., Hao Z., Lin J. (2022). Cutting deformation mechanism of SiCp/Al composites based on strain gradient theory. J. Mater. Process. Technol..

[22] Fan Y., Xu Y., Hao Z., Lin J. (2022). Dynamic behavior description and three-dimensional cutting simulation of SiCp/Al composites with high volume fraction. J. Manuf. Process..

[23] Li M., Li Q., Pan X., Wang J., Wang Z., Xu S., Zhou Y., Ma L., Yu T. (2025). Removal mechanism and damage evolution of SiCp/Al composites based on FEM-MD model considering 3D random polyhedral particles in orthogonal cutting. J. Mater. Res. Technol..

[24] Zhou Y., Liu J., Wang S., Chen H., Li D., Ma L., Li M. (2024). Study on the removal mechanism and milling quality of helical milling hole of SiCp/Al composites. J. Manuf. Process..

[25] Pramanik A., Zhang L.C. (2017). Particle fracture and debonding during orthogonal machining of metal matrix composites. Adv. Manuf..

[26] Sun W., Duan C., Yin W. (2021). Chip formation mechanism in machining of Al/SiCp composites based on analysis of particle damage. J. Manuf. Process..

[27] Liu C., Gao L., Jiang X., Xu W., Liu S., Yang T. (2020). Analytical modeling of subsurface damage depth in machining of SiCp/Al composites. Int. J. Mech. Sci..

[28] Chen D., Zheng Z., Wu D., Zeng C., Zang Y., She Z., Zhang J., Chen X., Xu J. (2025). Investigation on the machining mechanism and surface integrity in ultrasonic elliptical vibration cutting of Al-Si alloys. Precis. Eng..

[29] Varga J., Kender Š., Kaščák Ľ., Rohaľ V., Spišák E. (2024). Evaluation of non-planar tool interaction in milling of shaped surfaces using a copy milling cutter. Appl. Sci..

[30] Varga J., Demko M., Kaščák Ľ., Ižol P., Vrabeľ M., Brindza J. (2024). Influence of tool inclination and effective cutting speed on roughness parameters of machined shaped surfaces. Machines.

[31] Jing L., Niu Q., Yue W., Rong J., Gao H., Tang S. (2023). Groove bottom material removal mechanism and machinability evaluation for longitudinal ultrasonic vibration assisted milling of Al-50wt% Si alloy. Int. J. Adv. Manuf. Technol..

[32] Liu Y., Liang G., Gu Y., Lin J., Fu B., Gao T., Zhao J., Luan Y. (2025). Study on PCD tool wear in pulsed laser assisted turning Al-50wt% Si alloy. Mater. Today Commun..

[33] Yu W., Chen J., Ming W., An Q., Chen M. (2022). Feasibility of supercritical CO_2_-based minimum quantity lubrication to improve the surface integrity of 50% Sip/Al composites. J. Manuf. Process..

[34] Du Y., Wang W., Lu M., Lin J., Qiao Z. (2026). Investigation on removal mechanism transformation and subsurface microscopic evolution of SiCp/Al composites under ultrasonic elliptical vibration cutting. J. Mater. Res. Technol..

[35] Zhou J., Lu M., Lin J., Zhou X., Guo M., Du Y. (2022). Investigation of surface integrity transition of SiCp/Al composites based on specific cutting energy during ultrasonic elliptical vibration assisted cutting. J. Manuf. Process..

[36] Zuo C., Zhang H., Zhang J., Gao Z., Wang K. (2025). Research on machinability of 50% SiCp/Al with high-speed milling under supercritical carbon dioxide (scCO_2_)-based cooling conditions. J. Manuf. Process..

[37] Zhang H., Cui F., Yang M., Yan R., Peng F., Deng B., Lv J., Tang X. (2026). Investigation of surface quality consistency in laser-assisted milling of SiCp/Al composites under different laser preheating strategies. Opt. Laser Technol..

[38] Peng P., Tian X., Yu H., Bie W., Feng Y., Li C., Niu K., Xiang D., Gao G. (2026). Synergistic mechanism of laser-ultrasonic elliptical vibration on turning damage suppression in SiCp/Al composites. Eng. Fail. Anal..

[39] Yu B., Gu Y., Lin J., Zhang X., Wu H., Li S., Li G., Luan Y., Gao M. (2026). Synergistic effect of laser-induced plastic deformation and ultrasonic chip fracture on surface quality and tool life during SiCp/Al turning. Appl. Surf. Sci..

[40] Wang M., Zheng Y., Wu Z., Chen Z., Zhu Y., Zhang J., Xiao J., Xu J. (2026). Investigation on the material removal and damage suppression mechanisms of SiCp/Al composites by in-situ laser-assisted diamond cutting. Opt. Laser Technol..

[41] She Z., Liu C., Ke J., Zang Y., Zhang J., Chen X., Xu J. (2025). Material removal mechanism of cryogenic-laser assisted cutting for SiCp/Al composites. Opt. Laser Technol..

[42] Lazoglu I., Ulutan D., Alaca B.E., Engin S., Kaftanoglu B. (2008). An enhanced analytical model for residual stress prediction in machining. CIRP Ann.-Manuf. Technol..

[43] Saif M.T.A., Hui C.Y., Zehnder A.T. (1993). Interface shear stresses induced by nonuniform heating of a film on a substrate. Thin Solid Films.

[44] Huang Z., Zhang X., Yang C., Xiao B., Ma Z. (2018). Abnormal deformation behavior and particle distribution during hot compression of fine-grained 14vol% SiCp/2014Al composite. J. Alloys Compd..

[45] Dabade U.A., Joshi S.S. (2009). Analysis of chip formation mechanism in machining of Al/SiCp metal matrix composites. J. Mater. Process. Technol..

[46] Szwajka K., Zielińska-Szwajka J., Trzepieciński T. (2023). Improving the surface integrity of 316L steel in the context of bioimplant applications. Materials.

[47] El-Gallab M., Sklad M. (1998). Machining of Al/SiC particulate metal matrix composites-Part II: Workpiece surface integrity. J. Mater. Process. Technol..

[48] Pramanik A., Islam M.N., Davies I.J., Boswell B., Dong Y., Basak A.K., Uddin M.S., Dixit A.R., Chattopadhyaya S. (2017). Contribution of machining to the fatigue behaviour of metal matrix composites (MMCs) of varying reinforcement size. Int. J. Fatigue.

[49] Yuan S., Duan C., Liu Y., Yang L., Chen H. (2025). Mechanistic insights into the link between milling-induced surface layer particle damage and mechanical property evolution of SiCp/2009Al composite thin-walled parts. J. Mater. Process. Technol..

[50] Gao L., Liu C., Liu J., Yang T. (2023). Effect of subsurface damage on tensile behavior and fracture mechanism of SiCp/Al composites: Experimental analysis and RVE modeling. Eng. Fail. Anal..

